# Supplementary material for: Study protocol for the Multiple Symptoms Study 3: a pragmatic, randomised controlled trial of a clinic for patients with persistent (medically unexplained) physical symptoms
Source: BMJ Open. 2022 Nov 15;12(11):e066511. doi: 10.1136/bmjopen-2022-066511 (PMC9668014; doi:10.1136/bmjopen-2022-066511)
Supplement: Supplementary data [file bmjopen-2022-066511supp001.pdf]

Supplementary material 1.

Search Strategy for MSS3

The search involve 4 steps; participants must meet inclusion criteria at each of the 4 stages

1. Inclusion based on age
2. Inclusion based on having no codes for serious medical conditions listed at any time
3. Inclusion if at least one code for a symptom disorder / syndrome (or repeat prescription for one in the last 10 years)
4. Inclusion if at least 2 referrals for specialist care in last 3 years.

Codes listed are Read CTV2

1. Age >18 & <70

2. AND NONE, EVER, OF

   a. Cancer (B. excluding B7, B8, BB)

   b. Diabetes mellitus (C10)

   c. Schizophrenic disorders (E10)

   d. Parkinson's disease(F12)

   e. Ischaemic heart disease (G3)

   f. Heart failure (G58)

   g. Cerebrovascular disease (G6)

   h. Rheumatoid arthritis and other inflammatory polyarthropathy (N04)

   i. Senile and presenile organic psychotic conditions (E00)

   j. Alcoholic psychoses (E02)

   k. Drug psychoses (E04)

   l. Other chronic organic psychoses (E04)

   m. [X]Organic, including symptomatic, mental disorders (Eu0)

   n. Other cerebral degenerations (F11)

   o. Housebound (13CA)

   p. [V]Palliative care (Zv57C)

   q. Palliative treatment (8BJ1)

   r. Terminal illness (1Z0)

s. X]Mental retardation (Eu7)

t. Mental retardation (E3)

u. [X]Specific developmental disorders of scholastic skills (Eu81)

3. AND EITHER ONE OR MORE IN THE LAST 10 YEARS OF

a. Psychalgia (E278)

b. [X]Tension type headache (F2626)

c. [D]Facial pain (R0400)

d. Temporomandibular joint disorders (J046)

e. History of irritable bowel syndrome (14CF)

f. Other female genital symptom (K58y)

g. [D]Pelvic and perineal pain R090G

h. Fibromyalgia N239

i. Fibromyalgia N248

j. [D]Non cardiac chest pain R065B

k. [D]Chronic intractable pain R00zC

l. [X]Dissociative [conversion] disorders Eu44

m. [X]Somatoform disorders Eu45

n. [X]Organic dissociative disorder Eu055

o. Hysteria E201 (excluding E2019, E201B, E201C)

p. [X]Mixed dissociative [conversion] disorders Eu447

q. [X]Unsp behav synd assoc with physiol disturb physical facts Eu5z

r. Functional gastrointestinal tract disorders NEC J52 excluding (J522,J523,J524)

s. Non epilepsy attack disorder EMISNQNO78

t. Medically unexplained symptoms 16T

u. Psychogenic vomiting NOS E2754

v. Functional vomiting J16y5

w. Persistent vomiting J162

x. 302641015

y. Other specified stomach function disorders J16y (excluding J16y0, J16y1, J16y2, J16y3,J16y4, J16yz)

z. [X]Nonorganic dyspareunia Eu256

aa. Physiological malfunction arising from mental factors E26

bb. Dysequilibrium syndrome SP3y8

OR ONE OR MORE IN THE LAST 3 YEARS OF REPEAT PRESRIPTION ISSUED FOR

cc. Hysocine butyl bromide

dd. Dicycloverine Hydrochloride

ee. Mebeverine Hydrochloride

ff. Dicyclomine

gg. Alverine citrate

4. AND

EITHER

Choose and book referral (8Hp) (>= 2 in last 3 years)

OR

Other referrals (see codes) (>= 2 in last 3 years) from any of the following

- Referral to physician 8H4 [except derm (8H43, 8H4S), geri (8H47, 8H4D, LD psych 8H4f, vasectomy 8h4i);
- Referral to surgeon 8H5 (except neuro 8H55; obstetric 8H57; Plastic 8H59)
- Priority cancer referral 8Hn (except skin 8Hn0; Breast 8Hn2; Haem 8Hn6
