# Supplementary material for: Study protocol for the Multiple Symptoms Study 3: a pragmatic, randomised controlled trial of a clinic for patients with persistent (medically unexplained) physical symptoms
Source: BMJ Open. 2022 Nov 15;12(11):e066511. doi: 10.1136/bmjopen-2022-066511 (PMC9668014; doi:10.1136/bmjopen-2022-066511)
Supplement: Supplementary data [file bmjopen-2022-066511supp002.pdf]

Supplementary material 2.

The University Of Sheffield. Clinical Trials Research Unit.

Multiple Symptoms Study 3

IRAS ID: 232038

Ethics reference: 18/NW/0422

Participant Identification Number:

CONSENT FORM

Title of Project: Multiple Symptoms Study 3

Researcher reads the following statement: Before I enter you in to the trial, I need to confirm with you that you understand what is involved and whether or not you agree to take part. I will be recording this process. Is that all right with you? (If yes, start recording and proceed as follows, refer to Baseline appointment guidance if required)

Please can you confirm your name and the date [pause for response]. I am going to read some statements to you. After each one, please answer 'yes' if you agree with the statement; or, 'no' if you do not agree with the statement.

Enter response (Y/N)

1. I confirm that I have read the information sheet dated................... (version...........) for the above study. I have had the opportunity to consider the information, ask questions and have had these answered satisfactorily. Y/N

2. I understand that it is my choice to participate and that I am free to leave the study at any time without giving any reason, and without my medical care or legal rights being affected. Y/N

3. I understand that relevant sections of my medical notes and data collected during the study may be looked at by research staff involved in the study, from regulatory authorities or from the NHS Trust, where it is relevant to my taking part in this research. I give permission for these individuals to have access to my records. Y/N

4. I agree that the research team can use the information that I have provided in the completed questionnaire/s for the purposes of this study. Y/N

5. I agree that if I am allocated to receive the Symptoms Clinic that these consultations can be audio recorded. Y/N

6. I understand that the information collected about me may be used to support other research in the Y/N

Mooney C, *et al. BMJ Open* 2022; 12:e066511. doi: 10.1136/bmjopen-2022-066511

Supplementary material 2.

future, and may be shared anonymously with other researchers.

7. I agree to my General Practitioner being informed of my participation in the study and relevant information about me being exchanged between my GP and the research team. Y/N

8. I agree that a copy of this consent form and other study documents can be posted to the University of Sheffield for monitoring purposes Y/N

9. I agree to take part in the above study. Y/N

Optional Statements

10. I am happy for a member of the research team to contact me about taking part in an interview about my experience in this study. (Please note, this is entirely optional and does not affect your involvement in the rest of the study). Y/N

11. I agree that members of the research team can approach me at a later date to ask me if I wish to participate in future studies similar to this project. (Please note, this is entirely optional and does not affect your involvement in the rest of the study). Y/N

Declaration

I confirm that I have read each statement to the participant and they have given full consent to take part.

Name of person taking audio consent	Date	Signature

When completed: 1 for participant; 1 for researcher site file

MSS3 Consent form v3.0, 09.07.2020
