# Supplementary material for: Study protocol for the Multiple Symptoms Study 3: a pragmatic, randomised controlled trial of a clinic for patients with persistent (medically unexplained) physical symptoms
Source: BMJ Open. 2022 Nov 15;12(11):e066511. doi: 10.1136/bmjopen-2022-066511 (PMC9668014; doi:10.1136/bmjopen-2022-066511)
Supplement: Supplementary data [file bmjopen-2022-066511supp003.pdf]

Supplementary material 3.

The Symptoms Clinic Intervention

The Recognition, Explanation, Action and Learning (REAL) intervention was structured as follows.

Recognition takes place during the history-taking phase of the intervention. It includes explicit recognition of, and belief in, the reality and legitimacy of the patient's experience. It also includes explicit recognition that persistent physical symptoms are within the scope of these medical consultations and can be understood without recourse to primary psychological causes. Recognition also seeks to build therapeutic alliance between ER-GP and patient.

Explanation seeks to propose and negotiate explanations for symptoms in terms of body physiology, and sensory signal processing. Explanations seek to portray symptoms as understandable (in contrast to the idea of "medically unexplained symptoms") adaptive responses in body processes. ER-GPs delivering the Symptoms Clinic are encouraged to use the names of syndromes such as irritable bowel syndrome and fibromyalgia where criteria for these are met. However, explanations aim to provide mechanisms for the symptoms which extend beyond simply attributing a symptom to a syndrome.

Action to manage symptoms is proposed after explanations have been offered and negotiated. Actions can include attending to the body, thoughts and emotions, and the personal or social environment. Body-focused actions include breathing techniques (diaphragmatic breathing, slow paced breathing), relaxation, sensory grounding and simple guided imagery[20]. Actions around thoughts and emotions range include addressing catastrophic or symptom-focused thinking. Actions around behaviors include pacing, effective rest and behavioural activation (where dysphoria was an issue). For some patients recommended action includes taking steps to engage with psychological therapies – for instance where trauma emerges as part of the explanation.

Learning comes from the participant implementing agreed actions and evaluating the impact of them on their symptoms. Learning also relates to the importance of summing up sessions and the course of treatment with key take-homes. This is also facilitated by letters to the patient's usual GP (copied to the patient) after the first and final consultation summarizing some of the key points covered in the clinic.

The Symptoms Clinic intervention is described in a manual provided to the ER-GPs; however it is designed to be delivered flexibly and in a person-centred way which allows the clinician considerable freedom to focus on aspects of the patient's problem that they deem most appropriate and in forms of words they personally feel comfortable with.
