# Supplementary material for: Study protocol for the Multiple Symptoms Study 3: a pragmatic, randomised controlled trial of a clinic for patients with persistent (medically unexplained) physical symptoms
Source: BMJ Open. 2022 Nov 15;12(11):e066511. doi: 10.1136/bmjopen-2022-066511 (PMC9668014; doi:10.1136/bmjopen-2022-066511)
Supplement: Author's manuscript [file bmjopen-2022-066511.draft_revisions.pdf]

BMJ Open is committed to open peer review. As part of this commitment we make the peer review history of every article we publish publicly available.

When an article is published we post the peer reviewers' comments and the authors' responses online. We also post the versions of the paper that were used during peer review. These are the versions that the peer review comments apply to.

The versions of the paper that follow are the versions that were submitted during the peer review process. They are not the versions of record or the final published versions. They should not be cited or distributed as the published version of this manuscript.

BMJ Open is an open access journal and the full, final, typeset and author-corrected version of record of the manuscript is available on our site with no access controls, subscription charges or pay-per-view fees (http://bmjopen.bmj.com).

If you have any questions on BMJ Open's open peer review process please email info.bmjopen@bmj.com

BMJ Open

Study protocol for the Multiple Symptoms Study 3:A pragmatic, randomised controlled trial of a clinic for patients with persistent (medically unexplained) physical symptoms

Journal:	*BMJ Open*
Manuscript ID	bmjopen-2022-066511
Article Type:	Protocol
Date Submitted by the Author:	13-Jul-2022

Complete List of Authors:	Mooney, Cara; The University of Sheffield, School of Health and Related Research<br>White, David; University of Sheffield, Clinical Trials Research Unit<br>Dawson, Jeremy; The University of Sheffield, Sheffield University Management School<br>Deary, Vincent; Northumbria University, Psychology<br>Fryer, Kate; The University of Sheffield<br>Greco, Monica; Goldsmiths University of London<br>Horspool, Michelle; NHS Sheffield Clinical Commissioning Group<br>Neilson, Aileen; University of Edinburgh Division of Medical and Radiological Sciences,<br>Rowlands, Gillian; Newcastle University Institute for Health and Society,<br>Sanders, Tom; Northumbria University,<br>Thomas, Ruth; University of Aberdeen<br>Thomas, Steve; NHS Sheffield Clinical Commissioning Group<br>Waheed, Waquas; University of Manchester, Centre for Primary Care<br>Burton, Christopher; University of Sheffield, Academic Unit of Primary Medical Care

Keywords:	PRIMARY CARE, GENERAL MEDICINE (see Internal Medicine), INTERNAL MEDICINE

SCHOLARONE™
Manuscripts

Study protocol for the Multiple Symptoms Study 3: A pragmatic, randomised controlled trial of a clinic for patients with persistent (medically unexplained) physical symptoms

Protocol v5.2 07.07.2022

Mooney C[1], White D[1], Dawson J[2], Deary V[3], Fryer K[4], Greco M[5], Horspool M[6], Neilson AR[7], Rowlands G[8], Sanders T[9], Thomas RE[10], Thomas S[6], Waheed W[11] and Burton C[4]

Corresponding author

Cara Mooney

Clinical Trials Research Unit, School Health and Related Research,

University of Sheffield, Regent Court,

30 Regent Street,

Sheffield S1 4DA, UK,

Email: c.d.mooney@sheffield.ac.uk,

Telephone: +44 (0) 114 222 4308

For peer review only

For peer review only - http://bmjopen.bmj.com/site/about/guidelines.xhtml

Abstract

Introduction

Persistent physical symptoms (which cannot be adequately attributed to physical disease) affect around 1 million people (2% of adults) in the UK. They affect patients' quality of life and account for at least one third of referrals from GPs to specialists. These referrals give patients little benefit but have a real cost to health services time and diagnostic resources. The Symptoms Clinic has been designed to help people make sense of persistent physical symptoms (especially if medical tests have been negative) and to reduce the impact of symptoms on daily life.

Methods and analysis

This pragmatic, multi-centre, randomised controlled trial will assess the clinical and cost-effectiveness of the Symptoms Clinic intervention plus usual care compared with usual care alone. Patients were identified through GP searches and mail-outs and recruited by the central research team. 354 participants were recruited and individually randomised (1:1). The primary outcome is the self-reported PHQ-15 at 52 weeks post-randomisation. Secondary outcome measures include the EQ-5D-5L and health care resource use. Outcome measures will also be collected at 13 and 26 weeks post-randomisation. A process evaluation will be conducted including consultation content analysis and interviews with participants and key stakeholders.

Ethics and dissemination.

Ethics approval has been obtained via Greater Manchester Central Research Ethics Committee (Reference 18/NW/0422). The results of the trial will be submitted for publication in peer-reviewed journals, presented at relevant conferences and disseminated to trial participants and patient interest groups.

Trial Registration ISRCTN57050216

Strengths and limitations of this study, (up to five short bullet points, no longer than one sentence each, that relate specifically to the methods).

- The Symptoms Clinics are delivered by specially trained GPs in a structure that would allow broader roll out if shown to be effective
- Patients with lived experience were involved in the design of the trial and will provide advice throughout delivery
- Blinding of participants was not feasible due to the nature of the intervention
- Measures are taken to reduce the impact of this including blinding outcome data collectors and trial statisticians
- The embedded process evaluation will allow us to understand how the intervention works in practice and identify the processes underlying the outcomes

For peer review only

For peer review only - http://bmjopen.bmj.com/site/about/guidelines.xhtml

Introduction

Background and rationale

Persistent physical symptoms (PPS) which cannot be adequately attributed to physical disease affect approximately 1 million adults in the UK (2% of the adult population).[1 2] Many patients with such symptoms receive repeated referral and investigation[3] which provides little benefit [4] but has real costs to health services time and diagnostic resources.[5] When patients are told that medical tests do not show a cause for their symptoms they are commonly disappointed in their interactions with clinicians.[6 7] Patients want to have those symptoms explained in acceptable ways[8 9] in order to know that their symptoms are legitimate[6], to adapt to them and to manage them. Without an explanation for their symptoms many patients seek further healthcare use while at the same time losing confidence that it will help them. With acceptable explanation, patients may be able to move from looking for a cause, to self-managing their symptoms.[7]

PPS represents a broad category of disorders, including defined syndromes such as fibromyalgia or irritable bowel syndrome but also non-specific symptoms and combinations of symptoms from different syndromes.[10 11] The term replaces older and unhelpful terms including "medically unexplained symptoms" (MUS).[12] Recent thinking suggests that PPS, like chronic pain, should be regarded as disorders in their own right.[10] This fits with models of symptoms as consequences of disturbed interoception – the non-conscious sensing, interpreting and regulating the body.[13-15]

We developed a model of "rational explanation"[16] which enables clinicians to integrate knowledge from processes such as disturbed interoception, with patients' reported experiences, to develop explanations for symptoms. These rational explanations make sense of symptoms in terms of brain and body processes and are acceptable to doctor and patient.[17 18] They leave room for psychosocial influences without placing them as the cause, and they provide opportunities to guide self-management, which has been found to be of value to patients.[19] In rational explanations, psychological factors such as heightened vigilance to symptoms or persistent worry about symptoms are presented as understandable mechanisms by which symptoms persist rather than signs that symptoms have a "psychosomatic" cause. In contrast, previously advocated explanatory models such as somatisation are rejected by patients as too simplistic[8 9] and leave patients with PPS dissatisfied with the explanations they receive. Rational explanations based on signalling between the brain and the body also open up the possibility of using symptom management techniques which influence interoception and the autonomic nervous system including slow paced breathing.[20]

Improving PPS could have a substantial effect on health and on its impacts in terms of lost productivity and increased care needs. Physical symptoms not explained by disease account for very substantial costs[5] - between 40% and 60% of all referrals across a range of specialties,[4] estimated at £3bn annually to the NHS and £14bn to the wider economy.[21]

The Symptoms Clinic is a primary care intervention, designed to explore acceptable explanations for symptoms and to reduce the impact of PPS on daily life. The Multiple Symptoms Study 3 (MSS3),

For peer review only - http://bmjopen.bmj.com/site/about/guidelines.xhtml

randomised control trial (RCT), builds on successful preliminary studies which have shown the feasibility, and acceptability of the Symptoms Clinics.[22 23]

The primary aim of MSS3 is to determine the clinical and cost-effectiveness of the "Symptoms Clinic" intervention for patients with persistent ("medically unexplained") physical symptoms.

Objectives:

1. Conduct a pragmatic RCT, with internal pilot, of the Symptoms Clinic verses usual care, in people with PPS.
2. Establish Symptoms Clinics for the purposes of the trial, train Extended Role GPs (ER-GP) and provide them with supervision; systematically recruit patients from primary care, and ensure satisfactory trial procedures and follow-up.
3. Compare patient experience of physical symptoms and quality of life, as well as healthcare use, across 52 weeks, between participants allocated to the Symptom Clinic plus usual care and those allocated to usual care.
4. Understand the processes of change associated with the Symptoms Clinic by (a) conducting qualitative interviews with a subsample of participants (b) recording and coding key elements of the intervention, and (c) interviewing participants and stakeholders.

Methods and analysis

Trial design

MSS3 is a pragmatic, multi-centre, parallel group, individually randomised controlled trial, with internal pilot. It uses a superiority framework to compare the Symptoms Clinic intervention plus usual care to usual care alone.

Adaptations in response to the COVID-19 pandemic

The MSS3 RCT was originally designed and delivered as a face-to-face intervention. Prior to March 2020, enrolment appointments and delivery of the Symptoms Clinic took place in local GP practices or community research facilities. After a short pause due to COVID-19 restrictions the trial was re-designed to allow for remote delivery as described in this protocol. No changes were made to the content of the intervention. Sensitivity analyses will be conducted to explore differences in those receiving the intervention face-to-face and remotely, with a further sensitivity analysis removing those cases that were randomised immediately before the pause, for whom there was a substantial delay in the delivery of the Symptoms Clinic (so whose 13-week outcomes were sometimes collected before the intervention had begun; those randomised to the usual care group during the same period will also be removed for this sensitivity analysis). Qualitative interviews will explore participant and stakeholder opinion of the different delivery modalities.

Participants

Participants were recruited in four areas: Yorkshire and the Humber, Greater Manchester, Newcastle and

For peer review only - http://bmjopen.bmj.com/site/about/guidelines.xhtml
For peer review only

Gateshead, and Northwest London.

Inclusion and Exclusion criteria

Inclusion criteria:

1. Aged between 18 – 69 years (inclusive) at the time of the computer search
2. Current physical symptoms which meet the below criteria
    a. clinical records suggest PPS
    b. records show at least 2 referrals for specialist opinion in the last 36 months (extended to 42 months when restarting after the first pandemic wave)
    c. records show no evidence of any previous or current major illnesses likely to cause multiple symptoms
    d. doctors in the GP practice do not believe that the majority of the patient's symptoms can be currently explained by other pathology;
    e. the score on the PHQ-15 is between 10 and 20 (inclusive)
3. Access to a mobile phone with video calling capability or an email address and computer with video conferencing capability

Exclusion criteria:

1. A score of 3 on question 9 on the PHQ-9 completed at the enrolment appointment
2. Difficulty conducting a healthcare consultation in English without either a professional or family interpreter or other assistance
3. The GP regards inviting them to participate as inappropriate (e.g. recent bereavement)
4. Severe symptom-related disability (e.g. requiring help with daily personal care or severely impaired mobility)
5. Undergoing active multidisciplinary rehabilitation, IAPT programme or specialist psychological treatment including specialist pain, fatigue or other symptom clinic at the time of screening
6. Currently pregnant or less than 6 months postnatal at the time of the screening telephone call

A three-stage identification process was adopted: computer searching, GP record screening and postal invitation.

Computer searching

GP practices ran a computer search to identify patients. The search strategy is listed in supplementary materials 1.

GP record screening

A GP at the practice screened the list produced by the computer search to exclude patients for whom invitation may be inappropriate (e.g. major medical conditions not included in the search or concern about the appropriateness of invitation).

For peer review only - http://bmjopen.bmj.com/site/about/guidelines.xhtml

Postal invitation

The GP practices sent invitation packs containing an invitation letter, Participant Information Sheet (PIS), Physical Health Questionnaire-15 (PHQ-15) and a reply form with a pre-paid return envelope. Interested patients returned the reply form and the completed PHQ-15 to Sheffield CTRU. Reminder invitation packs were sent to non-responders approximately 3 weeks after the initial mailing. Respondents whose PHQ-15 was outside the eligible range were sent a letter and received no further contact.

Recruitment and informed consent

Potentially eligible patients, based on their PHQ-15 score, were contacted by the research team to provide further information and answer questions. If the patient wished to proceed with the study, the research team completed screening checks and if appropriate, scheduled a study enrolment appointment. During the enrolment appointment, a member of the recruitment team answered any final questions, obtained informed consent, confirmed eligibility and collected baseline data. Figure 1 presents the participant flow through the trial.

Randomisation and blinding

Following consent and baseline data collection, participants were individually randomised (1:1) to the Symptoms Clinic plus usual care or usual care alone, using a computer generated pseudo-random list, stratified by study centre with random permuted blocks of varying sizes. Allocation was concealed using a centralized web-based randomisation system.

The participant was then randomised and informed of their allocation. If assignment was to the intervention, the first Symptoms Clinic appointment was scheduled.

Due to the nature of the intervention, it is not possible to blind participants to their allocation. For practical reasons such as coordinating Symptoms Clinic appointments and ER-GP supervision some members of the research team are not blinded, including the Trial Manager and Chief Investigator (CI).

Members of the Trial Steering Committee (TSC), study statisticians, health economists and those collecting outcome data are blinded to treatment allocation while the trial is ongoing.

ER-GP Recruitment, Training and Supervision

Seven ER-GPs were recruited and trained to deliver the Symptoms Clinic. Two withdrew because of competing demands, one after seeing fewer than 5 patients and one before seeing any.

Training comprised a mixture of small group sessions (both didactic and interactive), protected time to conduct and reflect on symptom clinic consultation techniques in practice, and one-to-one or small group supervision. It involved 13 half-day sessions. Sessions 1-4 were two full days of training. Sessions 5-7 and 9-11 comprised protected time to see patients of the GPs own practice using newly learned skills and reflection on this. Sessions 8 and 12-13 were training sessions focusing on consolidating skills and

For peer review only - http://bmjopen.bmj.com/site/about/guidelines.xhtml
For peer review only

knowledge. In sessions 9-11 each ER-GP recorded a set of three consultations for review, quality assessment and constructive feedback by a panel comprising the CI and two other investigators.

During the study ER-GPs received supervision with one of the investigators approximately every 1 to 2 months. Supervision included review of consultation content and encouraged reflective learning and consolidation of existing knowledge and skills and learning of new knowledge and skills.

The Symptoms Clinic

The Symptoms Clinic intervention is a sequence of medical consultations which aim to elicit a detailed clinical history, ensure that the patient's experience is fully heard and validated, to offer rational explanations for symptoms and to assist the patient to develop ways of managing their symptoms. The treatment model can be summarized under four headings: Recognition, Explanation, Action and Learning (REAL). See supplementary materials 2 for further details.

Consultations before March 2020 were delivered face-to-face. Subsequently consultations took place via video consultation or telephone. The Symptoms Clinic consists of up to four consultations; an initial long consultation (approximately 50 minutes) followed by up to three medium length consultations (15-20 minutes) approximately every two weeks. Clinicians had flexibility to increase the gaps between sessions if required.

Fidelity of the Symptoms Clinic Intervention

All Symptoms Clinic consultations were audio-recorded. Approximately 1/3 are transcribed for quality assurance and process assessment and the remainder are archived for quality assurance purposes.

Fidelity is assessed from consultation transcripts or recordings against standards developed in the preliminary studies. The protocol originally proposed that this would include the proportion of consultation time spent on different components and the number and type of explanations. These proved difficult to operationalize and a simpler approach was adopted in which a framework of items in the intervention was used as a template and for each consultation the presence of each item was indicated and evidenced by using an extract or quote from the transcript. A traffic light system was used where clearly present was marked green, possibly present marked amber and absent marked red.

Symptoms Clinic attendance

Participants received appointment reminder text messages the day before each Symptoms Clinic appointment, which were personalized to include their name, ER-GP name, and appointment details. Attendance was monitored using the study database where re-arranged and missed appointments were recorded.

Outcomes

The primary outcome is the PHQ-15[24] at 52 weeks post-randomisation. The PHQ-15 consists of 15 items for

For peer review only - http://bmjopen.bmj.com/site/about/guidelines.xhtml

which patients are asked to report symptom severity over the past four weeks on a scale of 0 (not bothered at all), 1 (bothered a little) or 2 (bothered a lot). It has excellent internal reliability (α = 0.80) and good convergent validity with other measures of functionality, symptom severity and disability days[24].

The secondary outcomes are:

- European Quality of Life-5 Dimensions, 5 level version (EQ-5D-5L)[25]
- Patient Health Questionnaire-9 (PHQ-9)[26]
- Generalised Anxiety Disorder-7 (GAD-7)[27 28]
- SF-6D[29] derived from SF-12
- ICECAP-A[30 31]
- Patient Global Indicator of Change (PGIC)
- Ability to Participate in Social Roles and Activities (PROMIS)[32]
- Somatic Symptoms Disorder – B criteria scale (SSD-12)[33]
- European Health Literacy Survey (HLS EU-6)[34]
- Patient reported HCRU – a bespoke resource use questionnaire capturing healthcare use over the 52-week period, in primary and secondary care as well as NHS and private services.
- Medical note review of Healthcare Resource use (HCRU) - a bespoke resource use case report form (CRF) to capture the healthcare use over the 52-week period, in both primary and secondary care.

We are also collecting data on whether the participants have experienced symptoms of COVID-19.

Data Collection and Management

Self-report measures are collected by questionnaire at the enrolment appointment and by post at 13, 26 and 52 weeks post-randomisation. Non-responders are followed up. HCRU data will also be collected from primary care records. If primary care records cannot be accessed then the self-report questionnaire data will be used.

Researchers collecting and handling outcome measures will be blinded to participant allocation. The extraction of HCRU data from medical records will be completed after all other measures have been collected from the participant as it is possible that the outcome data collector will be unblinded through exposure to correspondence in the notes. The HCRU CRF will outline the order in which data is to be collected so correspondence is the last section to be reviewed.

If, at any stage, the outcome data collector know (or suspect) they have been unblinded, this will be recorded.

Data will be recorded in paper CRFs or online at the time of each participant contact. All CRFs use anonymised participant ID codes to protect confidentiality. Data is entered into Sheffield CTRU's web-based data management system (Prospect), by authorised members of the research team. All data are collected and retained in accordance with the Data Protection Act 2018, the General Data Protection Regulation and CTRU standard operating procedures (SOP).

Participant Retention

For peer review only - http://bmjopen.bmj.com/site/about/guidelines.xhtml

Participant retention is promoted through communication from the research team which clearly explains the importance of completing outcome data regardless of study arm. This message is reinforced at enrolment and all follow-up points. The questionnaire cover letter explains the importance of every returned questionnaire and participants are offered a £10 voucher on completion of the 52-week questionnaires.

Intervention completion and withdrawal

Intervention completion is defined as having an initial consultation and at least one follow-up consultation.

Participants may withdraw either from the intervention only or the trial and this is documented. If the participant withdraws from the trial, no further data will be collected.

Patient and Public Involvement

People with lived experience of PPS were involved in the design and development of MSS3. Patient participation was incorporated in the delivery of the project through representation in the Trial Management Group (TMG) and TSC.

Sample size

In the pilot trial we observed an average 3.2 point clinically important change in the intervention group from baseline to 13 weeks, compared to a 1.4 point change in the control group. We have thus powered the trial on a between group difference of 2 points on the PHQ-15 (equivalent to a clinically important 3 point change from baseline).

We have based calculations of effect size on a pooled standard deviation of 5; this is larger than that seen in our preliminary studies owing to their restricted eligibility range and more in keeping with observational studies. This results in a standardised effect size of 0.4, which is similar to that seen in two small European studies of extended GP consultations for broadly comparable patients.[35 36]

Calculation of sample size

Allowing 25% loss to follow-up, and a further pragmatic 6% inflation to allow for minor treatment centre imbalances or differences, a sample of 188 patients per arm has 90% power (alpha =0.05) to detect this effect. The initial recruitment target was thus 376 participants. In October 2021, this was reduced in discussion with the funder, to 350 because loss to follow-up at 52 weeks post-randomisation was 18% rather than the anticipated 25%.

Data Analysis

The primary outcome will be analysed using a partially nested heteroscedastic mixed-effects model to account for clustering by clinic GP. Secondary outcomes will be analysed in a similar manner within a generalised linear modelling framework using appropriate link functions for the outcomes' distributions. Models will adjust for sex, age, whether the intervention was delivered in person or online, and baseline

For peer review only - http://bmjopen.bmj.com/site/about/guidelines.xhtml
For peer review only

values of outcomes. A repeated measures analysis on PHQ-15 at all four measurement points will be conducted as a further secondary analysis using a multilevel growth curve model with time as a quadratic term, and a treatment-time interaction included in the model.

Intention to treat analysis will be used for the primary analysis of all outcomes, with complier average causal effect analysis as a secondary analysis. The primary outcome will be analysed using observed data with no imputation for missing data, but we will assess the amount and patterns of missing data and test the sensitivity of estimates of treatments effects using an appropriate imputation strategy such as multiple imputation by chained equation. We will explore potential modification of the treatment effect by including treatment-by-subgroup interactions in models. All treatment effect estimates will be presented with 95% confidence intervals in forest plots.

A single main analysis will be performed at the end of the trial when follow-up is complete. Interim analyses will be performed if requested by the Data Monitoring and Ethics Committee (DMEC) and CTRU SOPs will be adhered to maintain the integrity of the trial.

Process Evaluation

The process evaluation comprises three nested observational studies, including consultation content analysis and interviews with participants and key stakeholders.

Consultation content analysis

A sample of approximately 30% of consultations are transcribed. These will be used to examine the intervention content using the classification of consultation content, explanations and response to explanation which we have developed from the preliminary studies.[17 18 37] We will use this data to conduct exploratory analysis relating to explanation type, content and negotiation to patient outcomes in order to develop better understanding of the mechanisms by which the intervention affects outcomes.

Participant and stakeholder interviews

To explore processes of change within participants, semi-structured interviews are conducted with a purposive sample of participants at different stages of the intervention. Interviews will be transcribed and analysed thematically, recognising that there are likely to be changes in intra-personal understanding and interpretation (for which an interpretive phenomenological approach is likely to be valuable) and inter-personal or social understanding and interaction. Particular attention will be paid to patients' views on what aspects of the Symptoms Clinic were particularly valuable to them and how these translated into perceived changes in thoughts, behaviours and symptoms.

Stakeholder interviews will examine acceptability of the clinic concept and processes, skills learned and knowledge transferred, value for GPs and perceived value to patients.

Relationship between process evaluation and intervention delivery

MRC guidance on process evaluation highlights the importance of considering the relationship between

For peer review only - http://bmjopen.bmj.com/site/about/guidelines.xhtml

For peer review only

process evaluation and intervention delivery[38] including whether the process evaluation is allowed to inform the intervention or the two are independent of each other. Information was permitted to flow from the process evaluation to the intervention during the first three months of Symptoms Clinic delivery. These can be considered as the time of professional learning curves for both the ER-GPs and the supervising investigators. During this time early lessons can be learned and shared.

Health Economics

We will conduct a cost-effectiveness analysis (CEA) of the Symptoms Clinic plus usual care compared to usual care alone from the primary perspective of the UK NHS and Personal Social Services. This will be based on HCRU (including primary and secondary care contacts such as GP consultations, diagnostic tests and investigations, physical and mental health specialist referrals, and prescription psychotropic and pain-related medications) and outcome data collected during the trial. It will take the format of a within-trial CEA and use a cost-utility framework to estimate cost per Quality Adjusted Life Year (QALY) gained.

The effects of the intervention will be estimated as gain in QALYs at 52 weeks using health related quality of life data collected at baseline, 13, 26 and 52 weeks and the area under the curve method. Published UK tariffs will be used to convert these data to quality of life weights.

We will measure preference-based health-related quality of life using the EQ-5D-5L and the SF-6D. We will also use the newer capability wellbeing ICECAP-A measure to examine their relative responsiveness to change in this patient population.

A self-reported healthcare resource use questionnaire will be administered at 26 and 52-weeks post-randomisation to estimate healthcare resource use costs.

Data from GP electronic records at 52 weeks post-randomisation will be collected, where available and used for cross validation with self-reported data. Data from GP records will be extracted onto a standardised CRF.

Use of health care resources will be valued and the associated costs estimated by assigning unit costs from standard published UK sources (including Personal Social Service Research Unit (PSSRU) unit costs, NHS reference costs, British National Formulary (BNF)). Costs related to intervention delivery will be estimated using trial records, taking into account:

- face-to-face/video consultation clinic time,
- clinic-related administration
- clinician training,
- clinical supervision.

The CEA will be performed on an intention to treat basis (for participants with complete data on resource use and health utilities across all follow-up time points). The results of the analysis will be reported as incremental costs, effects and incremental cost-effectiveness ratios (ICERs) in terms of the incremental cost per QALY gained.

Generalised linear regression analyses will be used to estimate the differences (and associated 95% CIs) in per patient mean total costs and differences in mean total QALYs comparing the Symptoms Clinic

For peer review only - http://bmjopen.bmj.com/site/about/guidelines.xhtml

intervention plus usual care compared with usual care alone, adjusting for baseline differences in cost, utility and other patient characteristics (e.g. age, gender, PHQ-15 score). Uncertainty will be explored by conducting a range of one- and multi-way deterministic sensitivity analyses (or probabilistic sensitivity analysis if more appropriate) to test the robustness of the base case results including assuming a broader cost perspective (e.g. including private health care costs), evaluating the effect of missing values (comparing results based on complete cases and those estimated using multiple imputed values) and potential bias due to high-cost patients (removing these expensive participants from the analysis). Cost per QALY data will also be presented in the form of cost-effectiveness acceptability curves (CEAC) to show the probability that the intervention is cost effective for different values of willingness to pay per additional QALY.

Study Within a Trial (SWAT)

A SWAT will evaluate the impact of a pen and a brief PIS on levels of participant recruitment, using a factorial embedded RCT. Patients were randomised to: 1) A pen with the trial logo printed on, in addition to the standard invitation materials; 2) A pen with the trial logo printed on, in addition to a brief PIS, and the standard invitation materials; 3) A brief PIS, and the standard invitation materials; or 4) The standard invitation materials alone.

Ethics and dissemination

Safety

Adverse events (AEs) may be identified during participant consultations or from self-report measures. We will only collect AEs defined as 'expected' for this trial which include (a) significant exacerbation of mental distress defined as a PHQ-9 score of 20 or more and/or a score of 2 or 3 on question 9 (suicidality item), representing at least a 1 point score change (i.e a change from 2 to 3 from their previous measure), (b) self-harm, (c) emerging serious mental illness or substance use disorder identified after randomisation. All AEs which meet the definition of serious adverse event (SAE) will be collected and assessed for relatedness to the intervention. Related SAEs will be reported to the Sponsor and the Research Ethics Committee.

Governance

Sheffield CTRU on behalf of the Sponsor (NHS Sheffield Clinical Commissioning Group, 722 Prince of Wales Road, Darnall, Sheffield S9 4EU) coordinates the trial. The CI, project co-applicants, members of the data management team, Sponsor, Trial Manager and other representatives form the TMG, who oversee the operation of the trial. The TSC, comprised of two clinicians, a statistician, Health Economist and PPI representative, provides independent oversight. The independent DMEC comprised of two clinicians and a statistician reviews the trial data and advises the TSC on issues of patient safety and trial continuation.

Ethics approval

This trial was approved by Greater Manchester Central Research Ethics Committee, reference 18/NW/0422), on 25/06/2018.

For peer review only - http://bmjopen.bmj.com/site/about/guidelines.xhtml

Dissemination

We will publish the study's findings in peer-reviewed academic journals and present at local, national and international conferences where possible. We will publish a short summary of the results on the MSS3 website that can be accessed by all trial participants as well as relevant interest groups.

Author affiliations

[1]Clinical Trials Research Unit, University of Sheffield, Sheffield, UK

[2]School of Health and Related Research, The University of Sheffield, Sheffield, UK

[3]Department of Psychology, Northumbria University, Newcastle Upon Tyne, UK

[4]Academic Unit of Primary Medical Care, University of Sheffield, Sheffield, UK

[5]Department of Sociology, Goldsmiths, University of London, London, UK

[6]NHS Sheffield Clinical Commissioning Group, Sheffield, UK

[7]Edinburgh Clinical Trials Unit (ECTU), Usher Institute, University of Edinburgh, Edinburgh, UK

[8]Population Health Sciences Institute, Newcastle University, Newcastle, UK

[9]Department of Social Work, Education and Community Wellbeing, Northumbria University, Newcastle Upon Tyne, NE7 7XA, UK

[10]Centre for Healthcare Trials (CHaRT), Health Services Research Unit, University of Aberdeen, UK

[11]Centre for Primary care, Institute of Population Health, University of Manchester, Manchester, UK

Author contributions

CB, CM, DW, JD, VD, MG, MH, ARN, GR, TS, WW and RET cowrote the original trial protocol. CM lead the development of the protocol for publication and wrote the initial draft, KF, CB, TS and MG developed the process evaluation section. All authors contributed to reviewing and revising the draft versions prior to submission

Declaration of interests

None

Funding This study is funded by the NIHR Health Services and Delivery Research programme (project: 15/136/07). The views expressed in this publication are those of the authors and not necessarily those of the NHS, thee NIHR or the Department of Health.

Patient consent for publication Not required

For peer review only - http://bmjopen.bmj.com/site/about/guidelines.xhtml

References

1. Verhaak PF, Meijer SA, Visser AP, et al. Persistent presentation of medically unexplained symptoms in general practice. *Fam Pract* 2006;23(4):414-20. doi: 10.1093/fampra/cml016 [published Online First: 2006/04/25]
2. McGorm K, Burton C, Weller D, et al. Patients repeatedly referred to secondary care with symptoms unexplained by organic disease: prevalence, characteristics and referral pattern. *Family practice* 2010;27(5):479-86.
3. Nimnuan C, Hotopf M, Wessely S. Medically unexplained symptoms: an epidemiological study in seven specialities. *Journal of psychosomatic research* 2001;51(1):361-67.
4. Rolfe A, Burton C. Reassurance after diagnostic testing with a low pretest probability of serious disease: systematic review and meta-analysis. *JAMA internal medicine* 2013;173(6):407-16.
5. Burton C, McGorm K, Richardson G, et al. Healthcare costs incurred by patients repeatedly referred to secondary medical care with medically unexplained symptoms: a cost of illness study. *Journal of psychosomatic research* 2012;72(3):242-47. doi: 10.1016/j.jpsychores.2011.12.009
6. Nettleton S. 'I just want permission to be ill': towards a sociology of medically unexplained symptoms. *Soc Sci Med* 2006;62(5):1167-78. doi: 10.1016/j.socscimed.2005.07.030 [published Online First: 2005/09/02]
7. Johansen ML, Risor MB. What is the problem with medically unexplained symptoms for GPs? A meta-synthesis of qualitative studies. *Patient Educ Couns* 2017;100(4):647-54. doi: 10.1016/j.pec.2016.11.015 [published Online First: 2016/11/30]
8. Burbaum C, Stresing AM, Fritzsche K, et al. Medically unexplained symptoms as a threat to patients' identity? A conversation analysis of patients' reactions to psychosomatic attributions. *Patient Educ Couns* 2010;79(2):207-17. doi: 10.1016/j.pec.2009.09.043 [published Online First: 2009/11/17]
9. Peters S, Rogers A, Salmon P, et al. What do patients choose to tell their doctors? Qualitative analysis of potential barriers to reattributing medically unexplained symptoms. *J Gen Intern Med* 2009;24(4):443-9. doi: 10.1007/s11606-008-0872-x [published Online First: 2008/12/18]
10. Burton C, Fink P, Henningsen P, et al. Functional somatic disorders: discussion paper for a new common classification for research and clinical use. *BMC Med* 2020;18(1):34. doi: 10.1186/s12916-020-1505-4 [published Online First: 2020/03/04]
11. Rosendal M, Olde Hartman TC, Aamland A, et al. "Medically unexplained" symptoms and symptom disorders in primary care: prognosis-based recognition and classification. *BMC Fam Pract* 2017;18(1):18. doi: 10.1186/s12875-017-0592-6
12. Sharpe M. Somatic symptoms: beyond 'medically unexplained'. *Br J Psychiatry* 2013;203(5):320-1. doi: 10.1192/bjp.bp.112.122523 [published Online First: 2013/11/05]
13. Chen WG, Schloesser D, Arensdorf AM, et al. The Emerging Science of Interoception: Sensing, Integrating, Interpreting, and Regulating Signals within the Self. *Trends in Neurosciences* 2021;44(1):3-16. doi: 10.1016/j.tins.2020.10.007
14. Bonaz B, Lane RD, Oshinsky ML, et al. Diseases, Disorders, and Comorbidities of Interoception. *Trends in Neurosciences* 2021;44(1):39-51. doi: 10.1016/j.tins.2020.09.009
15. Henningsen P, Gundel H, Kop WJ, et al. Persistent Physical Symptoms as Perceptual Dysregulation: A Neuropsychobehavioral Model and Its Clinical Implications. *Psychosom Med* 2018;80(5):422-31. doi: 10.1097/psy.0000000000000588 [published Online First: 2018/04/06]
16. Burton C, Lucassen P, Aamland A, et al. Explaining symptoms after negative tests: towards a rational explanation. *J R Soc Med* 2015;108(3):84-8. doi: 10.1177/0141076814559082 [published Online First: 2014/11/13]
17. Morton L, Elliott A, Cleland J, et al. A taxonomy of explanations in a general practitioner clinic for patients with persistent "medically unexplained" physical symptoms. *Patient Educ Couns* 2017;100(2):224-30. doi: 10.1016/j.pec.2016.08.015
18. den Boeft M, Huisman D, Morton L, et al. Negotiating explanations: doctor-patient communication with patients with medically unexplained symptoms-a qualitative analysis. *Fam Pract* 2017;34(1):107-13. doi: 10.1093/fampra/cmw113 [published Online First: 2017/01/27]

For peer review only - http://bmjopen.bmj.com/site/about/guidelines.xhtml

19. Leaviss J, Davis S, Ren S, et al. Behavioural modification interventions for medically unexplained symptoms in primary care: systematic reviews and economic evaluation. *Health Technol Assess* 2020;24(46):1-490. doi: 10.3310/hta24460 [published Online First: 2020/09/26]
20. Weng HY, Feldman JL, Leggio L, et al. Interventions and Manipulations of Interoception. *Trends in Neurosciences* 2021;44(1):52-62. doi: 10.1016/j.tins.2020.09.010
21. Bermingham SL, Cohen A, Hague J, et al. The cost of somatisation among the working-age population in England for the year 2008–2009. *Mental health in Family medicine* 2010;7(2):71.
22. Burton C, Weller D, Marsden W, et al. A primary care Symptoms Clinic for patients with medically unexplained symptoms: pilot randomised trial. *BMJ Open* 2012;2(1):e000513. doi: 10.1136/bmjopen-2011-000513
23. Morton L, Elliott A, Thomas R, et al. Developmental study of treatment fidelity, safety and acceptability of a Symptoms Clinic intervention delivered by General Practitioners to patients with multiple medically unexplained symptoms. *J Psychosom Res* 2016;84:37-43. doi: 10.1016/j.jpsychores.2016.03.008 [published Online First: 2016/04/21]
24. Kroenke K, Spitzer RL, Williams JB. The PHQ-15: validity of a new measure for evaluating the severity of somatic symptoms. *Psychosom Med* 2002;64(2):258-66. doi: 10.1097/00006842-200203000-00008 [published Online First: 2002/03/27]
25. Herdman M, Gudex C, Lloyd A, et al. Development and preliminary testing of the new five-level version of EQ-5D (EQ-5D-5L). *Qual Life Res* 2011;20(10):1727-36. doi: 10.1007/s11136-011-9903-x [published Online First: 2011/04/12]
26. Kroenke K, Spitzer RL, Williams JB. The PHQ-9: validity of a brief depression severity measure. *J Gen Intern Med* 2001;16(9):606-13. doi: 10.1046/j.1525-1497.2001.016009606.x [published Online First: 2001/09/15]
27. Spitzer RL, Kroenke K, Williams JB, et al. A brief measure for assessing generalized anxiety disorder: the GAD-7. *Arch Intern Med* 2006;166(10):1092-7. doi: 10.1001/archinte.166.10.1092 [published Online First: 2006/05/24]
28. Kroenke K, Spitzer RL, Williams JB, et al. Anxiety disorders in primary care: prevalence, impairment, comorbidity, and detection. *Ann Intern Med* 2007;146(5):317-25. doi: 10.7326/0003-4819-146-5-200703060-00004 [published Online First: 2007/03/07]
29. Brazier J, Roberts J, Deverill M. The estimation of a preference-based measure of health from the SF-36. *Journal of Health Economics* 2002;21(2):271-92. doi: https://doi.org/10.1016/S0167-6296(01)00130-8
30. Simon J, Anand P, Gray A, et al. Operationalising the capability approach for outcome measurement in mental health research. *Soc Sci Med* 2013;98:187-96. doi: 10.1016/j.socscimed.2013.09.019 [published Online First: 2013/12/18]
31. Al-Janabi H, N Flynn T, Coast J. Development of a self-report measure of capability wellbeing for adults: the ICECAP-A. *Quality of Life Research* 2012;21(1):167-76. doi: 10.1007/s11136-011-9927-2
32. Hahn EA, DeWalt DA, Bode RK, et al. New English and Spanish social health measures will facilitate evaluating health determinants. *Health Psychol* 2014;33(5):490-9. doi: 10.1037/hea0000055 [published Online First: 2014/01/23]
33. Toussaint A, Löwe B, Brähler E, et al. The Somatic Symptom Disorder - B Criteria Scale (SSD-12): Factorial structure, validity and population-based norms. *J Psychosom Res* 2017;97:9-17. doi: 10.1016/j.jpsychores.2017.03.017 [published Online First: 2017/06/14]
34. Pelikan JM, Ganahl K. Measuring health literacy in general populations: Primary findings from the HLS-EU Consortium's health literacy assessment effort. *Stud Health Technol Inform* 2017;240:34-59.
35. Larisch A, Schweickhardt A, Wirsching M, et al. Psychosocial interventions for somatizing patients by the general practitioner: a randomized controlled trial. *J Psychosom Res* 2004;57(6):507-14; discussion 15-6. doi: 10.1016/j.jpsychores.2004.04.372 [published Online First: 2004/12/15]
36. Blankenstein AH, van der Horst HE, Schilte AF, et al. Development and feasibility of a modified reattribution model for somatising patients, applied by their own general practitioners. *Patient Educ Couns* 2002;47(3):229-35. doi: 10.1016/s0738-3991(01)00199-9 [published Online First: 2002/06/29]

For peer review only - http://bmjopen.bmj.com/site/about/guidelines.xhtml

37. Morton L, Elliott A, Thomas R, et al. Developmental study of treatment fidelity, safety and acceptability of a Symptoms Clinic intervention delivered by General Practitioners to patients with multiple medically unexplained symptoms. *Journal of Psychosomatic Research* 2016;84:37-43. doi: 10.1016/j.jpsychores.2016.03.008
38. Moore GF, Audrey S, Barker M, et al. Process evaluation of complex interventions: Medical Research Council guidance. *BMJ : British Medical Journal* 2015;350:h1258. doi: 10.1136/bmj.h1258

For peer review only

For peer review only - http://bmjopen.bmj.com/site/about/guidelines.xhtml

Figure Legend

Figure 1. Participant flow diagram

For peer review only

For peer review only - http://bmjopen.bmj.com/site/about/guidelines.xhtml

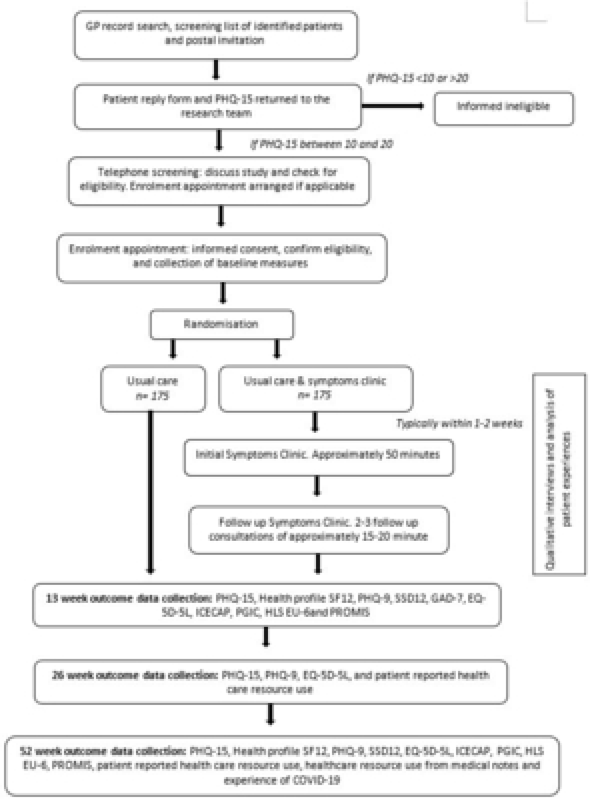

Figure 1. Participant flow diagram

12x17mm (600 x 600 DPI)

Supplementary material 1.

Search Strategy for MSS3

The search involve 4 steps; participants must meet inclusion criteria at each of the 4 stages

1. Inclusion based on age
2. Inclusion based on having no codes for serious medical conditions listed at any time
3. Inclusion if at least one code for a symptom disorder / syndrome (or repeat prescription for one in the last 10 years)
4. Inclusion if at least 2 referrals for specialist care in last 3 years.

Codes listed are Read CTV2

1. Age >18 & <70

2. AND NONE, EVER, OF

   a. Cancer (B. excluding B7, B8, BB)

   b. Diabetes mellitus (C10)

   c. Schizophrenic disorders (E10)

   d. Parkinson's disease(F12)

   e. Ischaemic heart disease (G3)

   f. Heart failure (G58)

   g. Cerebrovascular disease (G6)

   h. Rheumatoid arthritis and other inflammatory polyarthropathy (N04)

   i. Senile and presenile organic psychotic conditions (E00)

   j. Alcoholic psychoses (E02)

   k. Drug psychoses (E04)

   l. Other chronic organic psychoses (E04)

   m. [X]Organic, including symptomatic, mental disorders (Eu0)

   n. Other cerebral degenerations (F11)

   o. Housebound (13CA)

   p. [V]Palliative care (Zv57C)

   q. Palliative treatment (8BJ1)

   r. Terminal illness (1Z0)

For peer review only

s. X]Mental retardation (Eu7)

t. Mental retardation (E3)

u. [X]Specific developmental disorders of scholastic skills (Eu81)

3. AND EITHER ONE OR MORE IN THE LAST 10 YEARS OF

a. Psychalgia (E278)

b. [X]Tension type headache (F2626)

c. [D]Facial pain (R0400)

d. Temporomandibular joint disorders (J046)

e. History of irritable bowel syndrome (14CF)

f. Other female genital symptom (K58y)

g. [D]Pelvic and perineal pain R090G

h. Fibromyalgia N239

i. Fibromyalgia N248

j. [D]Non cardiac chest pain R065B

k. [D]Chronic intractable pain R00zC

l. [X]Dissociative [conversion] disorders Eu44

m. [X]Somatoform disorders Eu45

n. [X]Organic dissociative disorder Eu055

o. Hysteria E201 (excluding E2019, E201B, E201C)

p. [X]Mixed dissociative [conversion] disorders Eu447

q. [X]Unsp behav synd assoc with physiol disturb physical facts Eu5z

r. Functional gastrointestinal tract disorders NEC J52 excluding (J522,J523,J524)

s. Non epilepsy attack disorder EMISNQNO78

t. Medically unexplained symptoms 16T

u. Psychogenic vomiting NOS E2754

v. Functional vomiting J16y5

w. Persistent vomiting J162

For peer review only

x. 302641015

y. Other specified stomach function disorders J16y (excluding J16y0, J16y1, J16y2, J16y3,J16y4, J16yz)

z. [X]Nonorganic dyspareunia Eu256

aa. Physiological malfunction arising from mental factors E26

bb. Dysequilibrium syndrome SP3y8

OR ONE OR MORE IN THE LAST 3 YEARS OF REPEAT PRESRIPTION ISSUED FOR

cc. Hysocine butyl bromide

dd. Dicycloverine Hydrochloride

ee. Mebeverine Hydrochloride

ff. Dicyclomine

gg. Alverine citrate

4. AND

EITHER

Choose and book referral (8Hp) (>= 2 in last 3 years)

OR

Other referrals (see codes) (>= 2 in last 3 years) from any of the following

- Referral to physician 8H4 [except derm (8H43, 8H4S), geri (8H47, 8H4D, LD psych 8H4f, vasectomy 8h4i);
- Referral to surgeon 8H5 (except neuro 8H55; obstetric 8H57; Plastic 8H59)
- Priority cancer referral 8Hn (except skin 8Hn0; Breast 8Hn2; Haem 8Hn6

For peer review only

Supplementary material 2.

The Symptoms Clinic Intervention

The Recognition, Explanation, Action and Learning (REAL) intervention was structured as follows.

Recognition takes place during the history-taking phase of the intervention. It includes explicit recognition of, and belief in, the reality and legitimacy of the patient's experience. It also includes explicit recognition that persistent physical symptoms are within the scope of these medical consultations and can be understood without recourse to primary psychological causes. Recognition also seeks to build therapeutic alliance between ER-GP and patient.

Explanation seeks to propose and negotiate explanations for symptoms in terms of body physiology, and sensory signal processing. Explanations seek to portray symptoms as understandable (in contrast to the idea of "medically unexplained symptoms") adaptive responses in body processes. ER-GPs delivering the Symptoms Clinic are encouraged to use the names of syndromes such as irritable bowel syndrome and fibromyalgia where criteria for these are met. However, explanations aim to provide mechanisms for the symptoms which extend beyond simply attributing a symptom to a syndrome.

Action to manage symptoms is proposed after explanations have been offered and negotiated. Actions can include attending to the body, thoughts and emotions, and the personal or social environment. Body-focused actions include breathing techniques (diaphragmatic breathing, slow paced breathing), relaxation, sensory grounding and simple guided imagery[20]. Actions around thoughts and emotions range include addressing catastrophic or symptom-focused thinking. Actions around behaviors include pacing, effective rest and behavioural activation (where dysphoria was an issue). For some patients recommended action includes taking steps to engage with psychological therapies – for instance where trauma emerges as part of the explanation.

Learning comes from the participant implementing agreed actions and evaluating the impact of them on their symptoms. Learning also relates to the importance of summing up sessions and the course of treatment with key take-homes. This is also facilitated by letters to the patient's usual GP (copied to the patient) after the first and final consultation summarizing some of the key points covered in the clinic.

The Symptoms Clinic intervention is described in a manual provided to the ER-GPs; however it is designed to be delivered flexibly and in a person-centred way which allows the clinician considerable freedom to focus on aspects of the patient's problem that they deem most appropriate and in forms of words they personally feel comfortable with.

For peer review only

BMJ Open

Study protocol for the Multiple Symptoms Study 3:A pragmatic, randomised controlled trial of a clinic for patients with persistent (medically unexplained) physical symptoms

Journal:	*BMJ Open*
Manuscript ID	bmjopen-2022-066511.R1
Article Type:	Protocol
Date Submitted by the Author:	07-Sep-2022
Complete List of Authors:	Mooney, Cara; The University of Sheffield, School of Health and Related Research<br>White, David; University of Sheffield, Clinical Trials Research Unit<br>Dawson, Jeremy; The University of Sheffield, Sheffield University Management School<br>Deary, Vincent; Northumbria University, Psychology<br>Fryer, Kate; The University of Sheffield<br>Greco, Monica; Goldsmiths University of London<br>Horspool, Michelle; NHS Sheffield Clinical Commissioning Group<br>Neilson, Aileen; University of Edinburgh Division of Medical and Radiological Sciences,<br>Rowlands, Gillian; Newcastle University Institute for Health and Society,<br>Sanders, Tom; Northumbria University,<br>Thomas, Ruth; University of Aberdeen<br>Thomas, Steve; NHS Sheffield Clinical Commissioning Group<br>Waheed, Waquas; University of Manchester, Centre for Primary Care<br>Burton, Christopher; University of Sheffield, Academic Unit of Primary Medical Care
**Primary Subject Heading**:	General practice / Family practice
Secondary Subject Heading:	General practice / Family practice
Keywords:	PRIMARY CARE, GENERAL MEDICINE (see Internal Medicine), INTERNAL MEDICINE

SCHOLARONE™
Manuscripts

Study protocol for the Multiple Symptoms Study 3: A pragmatic, randomised controlled trial of a clinic for patients with persistent (medically unexplained) physical symptoms

Protocol v5.2 07.07.2022

Mooney C[1], White D[1], Dawson J[2], Deary V[3], Fryer K[4], Greco M[5], Horspool M[6], Neilson AR[7], Rowlands G[8], Sanders T[9], Thomas RE[10], Thomas S[6], Waheed W[11] and Burton C[4]

Corresponding author

Cara Mooney

Clinical Trials Research Unit, School Health and Related Research,

University of Sheffield, Regent Court,

30 Regent Street,

Sheffield S1 4DA, UK,

Email: c.d.mooney@sheffield.ac.uk,

Telephone: +44 (0) 114 222 4308

For peer review only - http://bmjopen.bmj.com/site/about/guidelines.xhtml

Abstract

Introduction

Persistent physical symptoms (which cannot be adequately attributed to physical disease) affect around 1 million people (2% of adults) in the UK. They affect patients' quality of life and account for at least one third of referrals from GPs to specialists. These referrals give patients little benefit but have a real cost to health services time and diagnostic resources. The Symptoms Clinic has been designed to help people make sense of persistent physical symptoms (especially if medical tests have been negative) and to reduce the impact of symptoms on daily life.

Methods and analysis

This pragmatic, multi-centre, randomised controlled trial will assess the clinical and cost-effectiveness of the Symptoms Clinic intervention plus usual care compared with usual care alone. Patients were identified through GP searches and mail-outs and recruited by the central research team. 354 participants were recruited and individually randomised (1:1). The primary outcome is the self-reported PHQ-15 at 52 weeks post-randomisation. Secondary outcome measures include the EQ-5D-5L and health care resource use. Outcome measures will also be collected at 13 and 26 weeks post-randomisation. A process evaluation will be conducted including consultation content analysis and interviews with participants and key stakeholders.

Ethics and dissemination.

Ethics approval has been obtained via Greater Manchester Central Research Ethics Committee (Reference 18/NW/0422). The results of the trial will be submitted for publication in peer-reviewed journals, presented at relevant conferences and disseminated to trial participants and patient interest groups.

Trial Registration ISRCTN57050216, registration date 02/10/2018

Strengths and limitations of this study, (up to five short bullet points, no longer than one sentence each, that relate specifically to the methods).

- The Symptoms Clinics are delivered by specially trained GPs in a structure that would allow broader roll out if shown to be effective
- Patients with lived experience were involved in the design of the trial and will provide advice throughout delivery
- Blinding of participants was not feasible due to the nature of the intervention
- Measures are taken to reduce the impact of this including blinding outcome data collectors and trial statisticians
- The embedded process evaluation will allow us to understand how the intervention works in practice and identify the processes underlying the outcomes

For peer review only

For peer review only - http://bmjopen.bmj.com/site/about/guidelines.xhtml

Introduction

Background and rationale

Persistent physical symptoms (PPS) which cannot be adequately attributed to physical disease affect approximately 1 million adults in the UK (2% of the adult population).[1 2] Many patients with such symptoms receive repeated referral and investigation[3] which provides little benefit [4] but has real costs to health services time and diagnostic resources.[5] When patients are told that medical tests do not show a cause for their symptoms they are commonly disappointed in their interactions with clinicians.[6 7] Patients want to have those symptoms explained in acceptable ways[8 9] in order to know that their symptoms are legitimate[6], to adapt to them and to manage them. Without an explanation for their symptoms many patients seek further healthcare use while at the same time losing confidence that it will help them. With acceptable explanation, patients may be able to move from looking for a cause, to self-managing their symptoms.[7]

PPS represents a broad category of disorders, including defined syndromes such as fibromyalgia or irritable bowel syndrome but also non-specific symptoms and combinations of symptoms from different syndromes.[10 11] The term replaces older and unhelpful terms including "medically unexplained symptoms" (MUS).[12] Recent thinking suggests that PPS, like chronic pain, should be regarded as disorders in their own right.[10] This fits with models of symptoms as consequences of disturbed interoception – the non-conscious sensing, interpreting and regulating the body.[13-15] Disturbed interoception, or more specifically disturbed predictive interoceptive coding,[15] provides a fundamental explanation for PPS which can then be elaborated in a number of psychological[16] and biopsychosocial[17] models for persistent symptoms. These often use frameworks such as predisposing, precipitating and persisting factors to structure explanations.

We developed a model of "rational explanation"[18] which enables clinicians to integrate knowledge from processes such as disturbed interoception, with patients' reported experiences, to develop explanations for symptoms. These rational explanations make sense of symptoms in terms of brain and body processes and are acceptable to doctor and patient.[19 20] They leave room for psychosocial influences without placing them as the cause, and they provide opportunities to guide self-management, which has been found to be of value to patients.[21] In rational explanations, psychological factors such as heightened vigilance to symptoms or persistent worry about symptoms are presented as understandable mechanisms by which symptoms persist rather than signs that symptoms have a "psychosomatic" cause. In contrast, previously advocated explanatory models such as somatisation are rejected by patients as too simplistic[8 9] and leave patients with PPS dissatisfied with the explanations they receive. Rational explanations based on signalling between the brain and the body also open up the possibility of using symptom management techniques which influence interoception and the autonomic nervous system including slow paced breathing.[22]

Improving PPS could have a substantial effect on health and on its impacts in terms of lost productivity and increased care needs. Physical symptoms not explained by disease account for very substantial costs[5] - between 40% and 60% of all referrals across a range of specialties,[4] estimated at £3bn annually to the NHS and £14bn to the wider economy.[23]

For peer review only - http://bmjopen.bmj.com/site/about/guidelines.xhtml
For peer review only

The Symptoms Clinic is a primary care intervention, designed to explore acceptable explanations for symptoms and to reduce the impact of PPS on daily life. The Multiple Symptoms Study 3 (MSS3), randomised control trial (RCT), builds on successful preliminary studies which have shown the feasibility, and acceptability of the Symptoms Clinics.[24 25]

The primary aim of MSS3 is to determine the clinical and cost-effectiveness of the "Symptoms Clinic" intervention for patients with persistent ("medically unexplained") physical symptoms.

Objectives:

1. Conduct a pragmatic RCT, with internal pilot, of the Symptoms Clinic verses usual care, in people with PPS.
2. Establish Symptoms Clinics for the purposes of the trial, train Extended Role GPs (ER-GP) and provide them with supervision; systematically recruit patients from primary care, and ensure satisfactory trial procedures and follow-up.
3. Compare patient experience of physical symptoms and quality of life, as well as healthcare use, across 52 weeks, between participants allocated to the Symptom Clinic plus usual care and those allocated to usual care.
4. Understand the processes of change associated with the Symptoms Clinic by (a) conducting qualitative interviews with a subsample of participants (b) recording and coding key elements of the intervention, and (c) interviewing stakeholders.

Methods and analysis

Trial design

MSS3 is a pragmatic, multi-centre, parallel group, individually randomised controlled trial, with internal pilot. It uses a superiority framework to compare the Symptoms Clinic intervention plus usual care to usual care alone.

Adaptations in response to the COVID-19 pandemic

The MSS3 RCT was originally designed and delivered as a face-to-face intervention. Prior to March 2020, enrolment appointments and delivery of the Symptoms Clinic took place in local GP practices or community research facilities. After a short pause due to COVID-19 restrictions the trial was re-designed to allow for remote delivery as described in this protocol. No changes were made to the content of the intervention. Sensitivity analyses will be conducted to explore differences in those receiving the intervention face-to-face and remotely, with a further sensitivity analysis removing those cases that were randomised immediately before the pause, for whom there was a substantial delay in the delivery of the Symptoms Clinic (so whose 13-week outcomes were sometimes collected before the intervention had begun; those randomised to the usual care group during the same period will also be removed for this sensitivity analysis). Qualitative interviews will explore participant and stakeholder opinion of the different delivery modalities.

For peer review only - http://bmjopen.bmj.com/site/about/guidelines.xhtml
For peer review only

Participants

Participants were recruited in four areas: Yorkshire and the Humber, Greater Manchester, Newcastle and Gateshead, and Northwest London.

Inclusion and Exclusion criteria

Inclusion criteria:

1. Aged between 18 – 69 years (inclusive) at the time of the computer search
2. Current physical symptoms which meet the below criteria
    a. clinical records suggest PPS
    b. records show at least 2 referrals for specialist opinion in the last 36 months (extended to 42 months when restarting after the first pandemic wave)
    c. records show no evidence of any previous or current major illnesses likely to cause multiple symptoms
    d. doctors in the GP practice do not believe that the majority of the patient's symptoms can be currently explained by other pathology;
    e. the score on the PHQ-15 is between 10 and 20 (inclusive)
3. Access to a mobile phone with video calling capability or an email address and computer with video conferencing capability

Exclusion criteria:

1. A score of 3 on question 9 on the PHQ-9 completed at the enrolment appointment
2. Difficulty conducting a healthcare consultation in English without either a professional or family interpreter or other assistance
3. The GP regards inviting them to participate as inappropriate (e.g. recent bereavement)
4. Severe symptom-related disability (e.g. requiring help with daily personal care or severely impaired mobility)
5. Undergoing active multidisciplinary rehabilitation, IAPT programme or specialist psychological treatment including specialist pain, fatigue or other symptom clinic at the time of screening
6. Currently pregnant or less than 6 months postnatal at the time of the screening telephone call

A three-stage identification process was adopted: computer searching, GP record screening and postal invitation.

Computer searching

GP practices ran a computer search to identify patients. The search strategy is listed in supplementary materials 1.

GP record screening

A GP at the practice screened the list produced by the computer search to exclude patients for whom invitation may be inappropriate (e.g. major medical conditions not included in the search or concern about

For peer review only - http://bmjopen.bmj.com/site/about/guidelines.xhtml

the appropriateness of invitation).

Postal invitation

The GP practices sent invitation packs containing an invitation letter, Participant Information Sheet (PIS), Physical Health Questionnaire-15 (PHQ-15) and a reply form with a pre-paid return envelope. Interested patients returned the reply form and the completed PHQ-15 to the clinical trials unit. Reminder invitation packs were sent to non-responders approximately 3 weeks after the initial mailing. Respondents whose PHQ-15 was outside the eligible range were sent a letter and received no further contact.

Recruitment and informed consent

Potentially eligible patients, based on their PHQ-15 score, were contacted by the research team to provide further information and answer questions. If the patient wished to proceed with the study, the research team completed screening checks and if appropriate, scheduled a study enrolment appointment. During the enrolment appointment, a member of the recruitment team answered any final questions, obtained informed consent (see supplementary materials 2), confirmed eligibility and collected baseline data. Figure 1 presents the participant flow through the trial.

Usual care

All participants continued to receive usual care from their registered general practice. While the intervention was delivered by specially trained GPs, this was done outside of their usual clinical practices and thus they had no contact with participants outside of the trial. The GP providing usual care was informed of their patients' participation and group allocation. GPs of patients allocated to the Symptoms Clinic also received a letter from the Symptoms Clinic doctor (copied to the participant) after the first and last appointments which outlined the formulation and any planned actions.

Randomisation and blinding

Following consent and baseline data collection, participants were individually randomised (1:1) to the Symptoms Clinic plus usual care or usual care alone, using a computer generated pseudo-random list, stratified by study centre with random permuted blocks of varying sizes. Allocation was concealed using a centralized web-based randomisation system.

The participant was then randomised and informed of their allocation. If assignment was to the intervention, the first Symptoms Clinic appointment was scheduled. If assignment was to usual care alone participants were reminded to use their usual healthcare services (e.g. usual care GP) as required.

Due to the nature of the intervention, it is not possible to blind participants to their allocation. For practical reasons such as coordinating Symptoms Clinic appointments and ER-GP supervision some members of the research team are not blinded, including the Trial Manager and Chief Investigator (CI).

Members of the Trial Steering Committee (TSC), study statisticians, health economists and those collecting

For peer review only - http://bmjopen.bmj.com/site/about/guidelines.xhtml

outcome data are blinded to treatment allocation while the trial is ongoing.

ER-GP Recruitment, Training and Supervision

Seven ER-GPs were recruited and trained to deliver the Symptoms Clinic. Two withdrew because of competing demands, one after seeing fewer than 5 patients and one before seeing any.

Training comprised a mixture of small group sessions (both didactic and interactive), protected time to conduct and reflect on symptom clinic consultation techniques in practice, and one-to-one or small group supervision. It involved 13 half-day sessions. Sessions 1-4 were two full days of training. Sessions 5-7 and 9-11 comprised protected time to see patients of the GPs own practice using newly learned skills and reflection on this. Sessions 8 and 12-13 were training sessions focusing on consolidating skills and knowledge. In sessions 9-11 each ER-GP recorded a set of three consultations for review, quality assessment and constructive feedback by a panel comprising the CI and two other investigators.

During the study ER-GPs received supervision with one of the investigators approximately every 1 to 2 months. Supervision included review of consultation content and encouraged reflective learning and consolidation of existing knowledge and skills and learning of new knowledge and skills.

The Symptoms Clinic

The Symptoms Clinic intervention is a sequence of medical consultations which aim to elicit a detailed clinical history, ensure that the patient's experience is fully heard and validated, to offer rational explanations for symptoms and to assist the patient to develop ways of managing their symptoms. The treatment model can be summarized under four headings: Recognition, Explanation, Action and Learning (REAL). See supplementary materials 3 for further details.

Consultations before March 2020 were delivered face-to-face. Subsequently consultations took place via video consultation or telephone. The Symptoms Clinic consists of up to four consultations; an initial long consultation (approximately 50 minutes) followed by up to three medium length consultations (15-20 minutes) approximately every two weeks. Clinicians had flexibility to increase the gaps between sessions if required.

Fidelity of the Symptoms Clinic Intervention

All Symptoms Clinic consultations were audio-recorded. A random sample of approximately 1/3 are transcribed for quality assurance and process assessment and the remainder are archived for quality assurance purposes. It was ensured that a sample from each ER-GP was selected.

Fidelity is assessed from consultation transcripts or recordings against standards developed in the preliminary studies. The protocol originally proposed that this would include the proportion of consultation time spent on different components and the number and type of explanations. These proved difficult to

For peer review only - http://bmjopen.bmj.com/site/about/guidelines.xhtml
For peer review only

operationalize and a simpler approach was adopted in which a framework of items in the intervention was used as a template and for each consultation the presence of each item was indicated and evidenced by using an extract or quote from the transcript. A traffic light system was used where clearly present was marked green, possibly present marked amber and absent marked red.

Symptoms Clinic attendance

Participants received appointment reminder text messages the day before each Symptoms Clinic appointment, which were personalized to include their name, ER-GP name, and appointment details. Attendance was monitored using the study database where re-arranged and missed appointments were recorded.

Outcomes

The primary outcome is the PHQ-15[26] at 52 weeks post-randomisation. The PHQ-15 consists of 15 items for which patients are asked to report symptom severity over the past four weeks on a scale of 0 (not bothered at all), 1 (bothered a little) or 2 (bothered a lot). It has excellent internal reliability ($\alpha = 0.80$) and good convergent validity with other measures of functionality, symptom severity and disability days[26].

The secondary outcomes are:

- Quality of Life measured using the EuroQol 5 dimension 5 level version (EQ-5D-5L).[27] In addition we will collect data for SF-6D[28] derived from SF-12 and ICECAP-A[29 30] to compare their performance in this study population
- Symptoms of depression and anxiety using the Patient Health Questionnaire-9 (PHQ-9)[31] and Generalised Anxiety Disorder-7 (GAD-7)[32 33]
- Healthcare resource use over the 52-week period using both self-report (a bespoke resource use questionnaire capturing healthcare use, in primary and secondary care as well as NHS and private services) and medical case note review of GP records.
- Patient reported Global Indicator of Change (PGIC)
- Ability to Participate in Social Roles and Activities (PROMIS)[34]

In addition, we will measure:

- Somatic Symptoms Disorder – B criteria scale (SSD-12)[35]
- European Health Literacy Survey (HLS EU-6)[36]

We are also collecting data on whether the participants have experienced symptoms of COVID-19.

Data Collection and Management

Self-report measures are collected by questionnaire at the enrolment appointment and by post at 13, 26 and 52 weeks post-randomisation. Non-responders are followed up.

Researchers collecting and handling outcome measures will be blinded to participant allocation. The extraction of healthcare use data from medical records will be completed after all other measures have been collected from the participant and in a pre-specifed order of extraction to minimize the risk of the outcome data collector becoming unblinded through exposure to correspondence in the notes before other data is collected.

For peer review only - http://bmjopen.bmj.com/site/about/guidelines.xhtml
For peer review only

If, at any stage, the outcome data collector know (or suspect) they have been unblinded, this will be recorded.

Data will be recorded in paper case report forms or online at the time of each participant contact. All forms use anonymised participant ID codes to protect confidentiality. Data is entered into the trial unit's web-based data management system (Prospect), by authorised members of the research team. All data are collected and retained in accordance with the Data Protection Act 2018, the General Data Protection Regulation and trial unit standard operating procedures (SOP).

Participant Retention

Participant retention is promoted through communication from the research team which clearly explains the importance of completing outcome data regardless of study arm. This message is reinforced at enrolment and all follow-up points. The questionnaire cover letter explains the importance of every returned questionnaire and participants are offered a £10 voucher on completion of the 52-week questionnaires.

Intervention completion and withdrawal

Intervention completion is defined as having an initial consultation and at least one follow-up consultation.

Participants may withdraw either from the intervention only or the trial and this is documented. If the participant withdraws from the trial, no further data will be collected.

Patient and Public Involvement

People with lived experience of PPS were involved in the design and development of MSS3. Patient participation was incorporated in the delivery of the project through representation in the Trial Management Group (TMG) and TSC.

Sample size

In the pilot trial we observed an average 3.2 point clinically important change in the intervention group from baseline to 13 weeks, compared to a 1.4 point change in the control group. We have thus powered the trial on a between group difference of 2 points on the PHQ-15 (equivalent to a clinically important 3 point change from baseline).

We have based calculations of effect size on a pooled standard deviation of 5; this is larger than that seen in our preliminary studies owing to their restricted eligibility range and more in keeping with observational studies. This results in a standardised effect size of 0.4, which is similar to that seen in two small European studies of extended GP consultations for broadly comparable patients.[37 38]

Calculation of sample size

Allowing 25% loss to follow-up, and a further pragmatic 6% inflation to allow for minor treatment centre imbalances or differences, a sample of 188 patients per arm has 90% power (alpha =0.05) to detect this

For peer review only - http://bmjopen.bmj.com/site/about/guidelines.xhtml

effect. The initial recruitment target was thus 376 participants. In October 2021, this was reduced in discussion with the funder, to 350 because loss to follow-up at 52 weeks post-randomisation was 18% rather than the anticipated 25%.

Data Analysis

The primary outcome will be analysed using a partially nested heteroscedastic mixed-effects model to account for clustering by clinic GP. Secondary outcomes will be analysed in a similar manner within a generalised linear modelling framework using appropriate link functions for the outcomes' distributions. Models will adjust for sex, age, whether the intervention was delivered in person or online, and baseline values of outcomes. A repeated measures analysis on PHQ-15 at all four measurement points will be conducted as a further secondary analysis using a multilevel growth curve model with time as a quadratic term, and a treatment-time interaction included in the model.
Intention to treat analysis will be used for the primary analysis of all outcomes, with complier average causal effect analysis as a secondary analysis. The primary outcome will be analysed using observed data with no imputation for missing data, but we will assess the amount and patterns of missing data and test the sensitivity of estimates of treatments effects using an appropriate imputation strategy such as multiple imputation by chained equation. We will explore potential modification of the treatment effect by including treatment-by-subgroup interactions in models. All treatment effect estimates will be presented with 95% confidence intervals in forest plots.

A single main analysis will be performed at the end of the trial when follow-up is complete. Interim analyses will be performed if requested by the Data Monitoring and Ethics Committee (DMEC) and the trials unit SOPs will be adhered to maintain the integrity of the trial.

Process Evaluation

The process evaluation comprises three nested observational studies, including consultation content analysis and interviews with participants and key stakeholders.

Consultation content analysis

A sample of approximately 30% of consultations are transcribed. These will be used to examine the intervention content using the classification of consultation content, explanations and response to explanation which we have developed from the preliminary studies.[19 20 39] We will use this data to conduct exploratory analysis relating to explanation type, content and negotiation to patient outcomes in order to develop better understanding of the mechanisms by which the intervention affects outcomes.

Participant and stakeholder interviews

To explore processes of change within participants, semi-structured interviews are conducted with a purposive sample of participants at different stages of the intervention. Only participants who did not have their consultations transcribed were invited for interview, and an even distribution was sought between patients with pain and fatigue type symptoms, and working and non-working patients. Interviews will be

For peer review only - http://bmjopen.bmj.com/site/about/guidelines.xhtml
For peer review only

transcribed and analysed thematically, recognising that there are likely to be changes in intra-personal understanding and interpretation (for which an interpretive phenomenological approach is likely to be valuable) and inter-personal or social understanding and interaction. Particular attention will be paid to patients' views on what aspects of the Symptoms Clinic were particularly valuable to them and how these translated into perceived changes in thoughts, behaviours and symptoms.

Stakeholder interviews will examine acceptability of the clinic concept and processes, skills learned and knowledge transferred, value for GPs and perceived value to patients.

Relationship between process evaluation and intervention delivery

MRC guidance on process evaluation highlights the importance of considering the relationship between process evaluation and intervention delivery[40] including whether the process evaluation is allowed to inform the intervention or the two are independent of each other. Information was permitted to flow from the process evaluation to the intervention during the first three months of Symptoms Clinic delivery. These can be considered as the time of professional learning curves for both the ER-GPs and the supervising investigators. During this time early lessons can be learned and shared.

Health Economics

We will conduct a cost-effectiveness analysis (CEA) of the Symptoms Clinic plus usual care compared to usual care alone from the primary perspective of the UK NHS and Personal Social Services. This will be based on HCRU (including primary and secondary care contacts such as GP consultations, diagnostic tests and investigations, physical and mental health specialist referrals, and prescription psychotropic and pain-related medications) and outcome data collected during the trial. It will take the format of a within-trial CEA and use a cost-utility framework to estimate cost per Quality Adjusted Life Year (QALY) gained.
The effects of the intervention will be estimated as gain in QALYs at 52 weeks using health related quality of life data collected at baseline, 13, 26 and 52 weeks and the area under the curve method. Published UK tariffs will be used to convert these data to quality of life weights.

We will measure preference-based health-related quality of life using the EQ-5D-5L and the SF-6D. We will also use the newer capability wellbeing ICECAP-A measure to examine their relative responsiveness to change in this patient population.

A self-reported healthcare resource use questionnaire will be administered at 26 and 52-weeks post-randomisation to estimate healthcare resource use costs.
Data from GP electronic records at 52 weeks post-randomisation will be collected, where available and used for cross validation with self-reported data. Data from GP records will be extracted onto a standardised data collection form.

Use of health care resources will be valued and the associated costs estimated by assigning unit costs from standard published UK sources (including Personal Social Service Research Unit (PSSRU) unit costs, NHS

For peer review only - http://bmjopen.bmj.com/site/about/guidelines.xhtml

reference costs, British National Formulary (BNF)). Costs related to intervention delivery will be estimated using trial records, taking into account:

- face-to-face/video consultation clinic time,
- clinic-related administration
- clinician training,
- clinical supervision.

The CEA will be performed on an intention to treat basis (for participants with complete data on resource use and health utilities across all follow-up time points). The results of the analysis will be reported as incremental costs, effects and incremental cost-effectiveness ratios (ICERs) in terms of the incremental cost per QALY gained.

Generalised linear regression analyses will be used to estimate the differences (and associated 95% CIs) in per patient mean total costs and differences in mean total QALYs comparing the Symptoms Clinic intervention plus usual care compared with usual care alone, adjusting for baseline differences in cost, utility and other patient characteristics (e.g. age, gender, PHQ-15 score). Uncertainty will be explored by conducting a range of one- and multi-way deterministic sensitivity analyses (or probabilistic sensitivity analysis if more appropriate) to test the robustness of the base case results including assuming a broader cost perspective (e.g. including private health care costs), evaluating the effect of missing values (comparing results based on complete cases and those estimated using multiple imputed values) and potential bias due to high-cost patients (removing these expensive participants from the analysis). Cost per QALY data will also be presented in the form of cost-effectiveness acceptability curves (CEAC) to show the probability that the intervention is cost effective for different values of willingness to pay per additional QALY.

Study Within a Trial (SWAT)

A SWAT will evaluate the impact of a pen and a brief PIS on levels of participant recruitment, using a factorial embedded RCT. Patients were randomised to: 1) A pen with the trial logo printed on, in addition to the standard invitation materials; 2) A pen with the trial logo printed on, in addition to a brief PIS, and the standard invitation materials; 3) A brief PIS, and the standard invitation materials; or 4) The standard invitation materials alone.

Ethics and dissemination

Safety

Adverse events (AEs) may be identified during participant consultations or from self-report measures. We will only collect AEs defined as ‘expected’ for this trial which include (a) significant exacerbation of mental distress defined as a PHQ-9 score of 20 or more and/or a score of 2 or 3 on question 9 (suicidality item), representing at least a 1 point score change (i.e a change from 2 to 3 from their previous measure), (b) self-harm, (c) emerging serious mental illness or substance use disorder identified after randomisation. All AEs which meet the definition of serious adverse event (SAE) will be collected and assessed for relatedness to the intervention. Related SAEs will be reported to the Sponsor and the Research Ethics Committee.

For peer review only - http://bmjopen.bmj.com/site/about/guidelines.xhtml

Governance

Sheffield Clinical Trials Research Unit (CTRU) on behalf of the Sponsor (NHS Sheffield Clinical Commissioning Group, 722 Prince of Wales Road, Darnall, Sheffield S9 4EU) coordinates the trial. The CI, project co-applicants, members of the data management team, Sponsor, Trial Manager and other representatives form the TMG, who oversee the operation of the trial and through which amendments will be communicated. The TSC, comprised of two clinicians, a statistician, Health Economist and PPI representative, provides independent oversight. The independent DMEC comprised of two clinicians and a statistician reviews the trial data and advises the TSC on issues of patient safety and trial continuation.

Ethics approval

This trial was approved by Greater Manchester Central Research Ethics Committee, reference 18/NW/0422), on 25/06/2018. The committee will be notified of any amendments to the trial protocol as appropriate.

Dissemination

We will publish the study's findings in peer-reviewed academic journals and present at local, national and international conferences where possible. We will publish a short summary of the results on the MSS3 website that can be accessed by all trial participants as well as relevant interest groups.

Data Sharing statement

Data are available upon reasonable request.

MSS3 Trial Data will not be archived in a repository, instead data will be released on a case-by-case basis. Data access requests will be reviewed and authorised by a subcommittee of the TMG during the trial and by the Sheffield CTRU after the trial has ended. Requests for data access should be made to Professor Chris Burton (ORCID 0000-0003-0233-2431). Data sharing will only take place if this aligns with the consent provided by MSS3 participants and the researchers provide a methodologically sound proposal. Data can only be used to achieve the aims in the approved proposal. Data will be anonymised prior to being shared. The Statistical Analysis Plan will be made freely available.

For peer review only

Author affiliations

[1]Clinical Trials Research Unit, University of Sheffield, Sheffield, UK

[2]School of Health and Related Research, The University of Sheffield, Sheffield, UK

[3]Department of Psychology, Northumbria University, Newcastle Upon Tyne, UK

[4]Academic Unit of Primary Medical Care, University of Sheffield, Sheffield, UK

[5]Department of Sociology, Goldsmiths, University of London, London, UK

[6]NHS Sheffield Clinical Commissioning Group, Sheffield, UK

For peer review only - http://bmjopen.bmj.com/site/about/guidelines.xhtml

[7]Edinburgh Clinical Trials Unit (ECTU), Usher Institute, University of Edinburgh, Edinburgh, UK

[8]Population Health Sciences Institute, Newcastle University, Newcastle, UK

[9]Department of Social Work, Education and Community Wellbeing, Northumbria University, Newcastle Upon Tyne, NE7 7XA, UK

[10]Centre for Healthcare Trials (CHaRT), Health Services Research Unit, University of Aberdeen, UK

[11]Centre for Primary care, Institute of Population Health, University of Manchester, Manchester, UK

Author contributions

CB, CM, DW, JD, VD, MG, MH, ARN, GR, TS, WW and RET cowrote the original trial protocol. CM lead the development of the protocol for publication and wrote the initial draft, KF, CB, TS and MG developed the process evaluation section. All authors contributed to reviewing and revising the draft versions prior to submission

Declaration of interests

None

Funding This study is funded by the NIHR Health Services and Delivery Research programme (project: 15/136/07). The views expressed in this publication are those of the authors and not necessarily those of the NHS, the NIHR or the Department of Health.

Patient consent for publication Not required

For peer review only - http://bmjopen.bmj.com/site/about/guidelines.xhtml

For peer review only

References

1. Verhaak PF, Meijer SA, Visser AP, et al. Persistent presentation of medically unexplained symptoms in general practice. *Fam Pract* 2006;23(4):414-20. doi: 10.1093/fampra/cml016 [published Online First: 2006/04/25]
2. McGorm K, Burton C, Weller D, et al. Patients repeatedly referred to secondary care with symptoms unexplained by organic disease: prevalence, characteristics and referral pattern. *Family practice* 2010;27(5):479-86.
3. Nimnuan C, Hotopf M, Wessely S. Medically unexplained symptoms: An epidemiological study in seven specialities. *Journal of Psychosomatic Research* 2001;51(1):361-67. doi: https://doi.org/10.1016/S0022-3999(01)00223-9
4. Rolfe A, Burton C. Reassurance after diagnostic testing with a low pretest probability of serious disease: systematic review and meta-analysis. *JAMA internal medicine* 2013;173(6):407-16.
5. Burton C, McGorm K, Richardson G, et al. Healthcare costs incurred by patients repeatedly referred to secondary medical care with medically unexplained symptoms: a cost of illness study. *Journal of psychosomatic research* 2012;72(3):242-47. doi: 10.1016/j.jpsychores.2011.12.009
6. Nettleton S. 'I just want permission to be ill': towards a sociology of medically unexplained symptoms. *Soc Sci Med* 2006;62(5):1167-78. doi: 10.1016/j.socscimed.2005.07.030 [published Online First: 2005/09/02]
7. Johansen ML, Risor MB. What is the problem with medically unexplained symptoms for GPs? A meta-synthesis of qualitative studies. *Patient Educ Couns* 2017;100(4):647-54. doi: 10.1016/j.pec.2016.11.015 [published Online First: 2016/11/30]
8. Burbaum C, Stresing AM, Fritzsche K, et al. Medically unexplained symptoms as a threat to patients' identity? A conversation analysis of patients' reactions to psychosomatic attributions. *Patient Educ Couns* 2010;79(2):207-17. doi: 10.1016/j.pec.2009.09.043 [published Online First: 2009/11/17]
9. Peters S, Rogers A, Salmon P, et al. What do patients choose to tell their doctors? Qualitative analysis of potential barriers to reattributing medically unexplained symptoms. *J Gen Intern Med* 2009;24(4):443-9. doi: 10.1007/s11606-008-0872-x [published Online First: 2008/12/18]
10. Burton C, Fink P, Henningsen P, et al. Functional somatic disorders: discussion paper for a new common classification for research and clinical use. *BMC Med* 2020;18(1):34. doi: 10.1186/s12916-020-1505-4 [published Online First: 2020/03/04]
11. Rosendal M, Olde Hartman TC, Aamland A, et al. "Medically unexplained" symptoms and symptom disorders in primary care: prognosis-based recognition and classification. *BMC Fam Pract* 2017;18(1):18. doi: 10.1186/s12875-017-0592-6
12. Sharpe M. Somatic symptoms: beyond 'medically unexplained'. *Br J Psychiatry* 2013;203(5):320-1. doi: 10.1192/bjp.bp.112.122523 [published Online First: 2013/11/05]
13. Bonaz B, Lane RD, Oshinsky ML, et al. Diseases, Disorders, and Comorbidities of Interoception. *Trends in Neurosciences* 2021;44(1):39-51. doi: 10.1016/j.tins.2020.09.009
14. Henningsen P, Gundel H, Kop WJ, et al. Persistent Physical Symptoms as Perceptual Dysregulation: A Neuropsychobehavioral Model and Its Clinical Implications. *Psychosom Med* 2018;80(5):422-31. doi: 10.1097/psy.0000000000000588 [published Online First: 2018/04/06]
15. Barrett LF, Simmons WK. Interoceptive predictions in the brain. *Nat Rev Neurosci* 2015;16(7):419-29. doi: 10.1038/nrn3950 [published Online First: 2015/05/29]
16. Deary V, Chalder T, Sharpe M. The cognitive behavioural model of medically unexplained symptoms: a theoretical and empirical review. *Clin Psychol Rev* 2007;27(7):781-97. doi: 10.1016/j.cpr.2007.07.002 [published Online First: 2007/09/08]
17. Löwe B, Andresen V, Van den Bergh O, et al. Persistent SOMAtic symptoms ACROSS diseases - from risk factors to modification: scientific framework and overarching protocol of the interdisciplinary SOMACROSS research unit (RU 5211). *BMJ Open* 2022;12(1):e057596. doi: 10.1136/bmjopen-2021-057596 [published Online First: 2022/01/23]
18. Burton C, Lucassen P, Aamland A, et al. Explaining symptoms after negative tests: towards a rational explanation. *J R Soc Med* 2015;108(3):84-8. doi: 10.1177/0141076814559082 [published Online First: 2014/11/13]

For peer review only - http://bmjopen.bmj.com/site/about/guidelines.xhtml

19. Morton L, Elliott A, Cleland J, et al. A taxonomy of explanations in a general practitioner clinic for patients with persistent "medically unexplained" physical symptoms. *Patient Educ Couns* 2017;100(2):224-30. doi: 10.1016/j.pec.2016.08.015
20. den Boeft M, Huisman D, Morton L, et al. Negotiating explanations: doctor-patient communication with patients with medically unexplained symptoms-a qualitative analysis. *Fam Pract* 2017;34(1):107-13. doi: 10.1093/fampra/cmw113 [published Online First: 2017/01/27]
21. Leaviss J, Davis S, Ren S, et al. Behavioural modification interventions for medically unexplained symptoms in primary care: systematic reviews and economic evaluation. *Health Technol Assess* 2020;24(46):1-490. doi: 10.3310/hta24460 [published Online First: 2020/09/26]
22. Weng HY, Feldman JL, Leggio L, et al. Interventions and Manipulations of Interoception. *Trends in Neurosciences* 2021;44(1):52-62. doi: 10.1016/j.tins.2020.09.010
23. Bermingham SL, Cohen A, Hague J, et al. The cost of somatisation among the working-age population in England for the year 2008–2009. *Mental health in Family medicine* 2010;7(2):71.
24. Burton C, Weller D, Marsden W, et al. A primary care Symptoms Clinic for patients with medically unexplained symptoms: pilot randomised trial. *BMJ Open* 2012;2(1):e000513. doi: 10.1136/bmjopen-2011-000513
25. Morton L, Elliott A, Thomas R, et al. Developmental study of treatment fidelity, safety and acceptability of a Symptoms Clinic intervention delivered by General Practitioners to patients with multiple medically unexplained symptoms. *J Psychosom Res* 2016;84:37-43. doi: 10.1016/j.jpsychores.2016.03.008 [published Online First: 2016/04/21]
26. Kroenke K, Spitzer RL, Williams JB. The PHQ-15: validity of a new measure for evaluating the severity of somatic symptoms. *Psychosom Med* 2002;64(2):258-66. doi: 10.1097/00006842-200203000-00008 [published Online First: 2002/03/27]
27. Herdman M, Gudex C, Lloyd A, et al. Development and preliminary testing of the new five-level version of EQ-5D (EQ-5D-5L). *Qual Life Res* 2011;20(10):1727-36. doi: 10.1007/s11136-011-9903-x [published Online First: 2011/04/12]
28. Brazier J, Roberts J, Deverill M. The estimation of a preference-based measure of health from the SF-36. *Journal of Health Economics* 2002;21(2):271-92. doi: https://doi.org/10.1016/S0167-6296(01)00130-8
29. Simon J, Anand P, Gray A, et al. Operationalising the capability approach for outcome measurement in mental health research. *Soc Sci Med* 2013;98:187-96. doi: 10.1016/j.socscimed.2013.09.019 [published Online First: 2013/12/18]
30. Al-Janabi H, N Flynn T, Coast J. Development of a self-report measure of capability wellbeing for adults: the ICECAP-A. *Quality of Life Research* 2012;21(1):167-76. doi: 10.1007/s11136-011-9927-2
31. Kroenke K, Spitzer RL, Williams JB. The PHQ-9: validity of a brief depression severity measure. *J Gen Intern Med* 2001;16(9):606-13. doi: 10.1046/j.1525-1497.2001.016009606.x [published Online First: 2001/09/15]
32. Spitzer RL, Kroenke K, Williams JB, et al. A brief measure for assessing generalized anxiety disorder: the GAD-7. *Arch Intern Med* 2006;166(10):1092-7. doi: 10.1001/archinte.166.10.1092 [published Online First: 2006/05/24]
33. Kroenke K, Spitzer RL, Williams JB, et al. Anxiety disorders in primary care: prevalence, impairment, comorbidity, and detection. *Ann Intern Med* 2007;146(5):317-25. doi: 10.7326/0003-4819-146-5-200703060-00004 [published Online First: 2007/03/07]
34. Hahn EA, DeWalt DA, Bode RK, et al. New English and Spanish social health measures will facilitate evaluating health determinants. *Health Psychol* 2014;33(5):490-9. doi: 10.1037/hea0000055 [published Online First: 2014/01/23]
35. Toussaint A, Löwe B, Brähler E, et al. The Somatic Symptom Disorder - B Criteria Scale (SSD-12): Factorial structure, validity and population-based norms. *J Psychosom Res* 2017;97:9-17. doi: 10.1016/j.jpsychores.2017.03.017 [published Online First: 2017/06/14]
36. Pelikan JM, Ganahl K. Measuring health literacy in general populations: Primary findings from the HLS-EU Consortium's health literacy assessment effort. *Stud Health Technol Inform* 2017;240:34-59.
37. Larisch A, Schweickhardt A, Wirsching M, et al. Psychosocial interventions for somatizing patients by the general practitioner: a randomized controlled trial. *J Psychosom Res* 2004;57(6):507-14; discussion 15-6. doi: 10.1016/j.jpsychores.2004.04.372 [published Online First: 2004/12/15]

For peer review only - http://bmjopen.bmj.com/site/about/guidelines.xhtml

38. Blankenstein AH, van der Horst HE, Schilte AF, et al. Development and feasibility of a modified reattribution model for somatising patients, applied by their own general practitioners. *Patient Educ Couns* 2002;47(3):229-35. doi: 10.1016/s0738-3991(01)00199-9 [published Online First: 2002/06/29]
39. Morton L, Elliott A, Thomas R, et al. Developmental study of treatment fidelity, safety and acceptability of a Symptoms Clinic intervention delivered by General Practitioners to patients with multiple medically unexplained symptoms. *Journal of Psychosomatic Research* 2016;84:37-43. doi: 10.1016/j.jpsychores.2016.03.008
40. Moore GF, Audrey S, Barker M, et al. Process evaluation of complex interventions: Medical Research Council guidance. *BMJ : British Medical Journal* 2015;350:h1258. doi: 10.1136/bmj.h1258

For peer review only

For peer review only - http://bmjopen.bmj.com/site/about/guidelines.xhtml

Figure Legend

Figure 1. Participant flow diagram

For peer review only

For peer review only - http://bmjopen.bmj.com/site/about/guidelines.xhtml

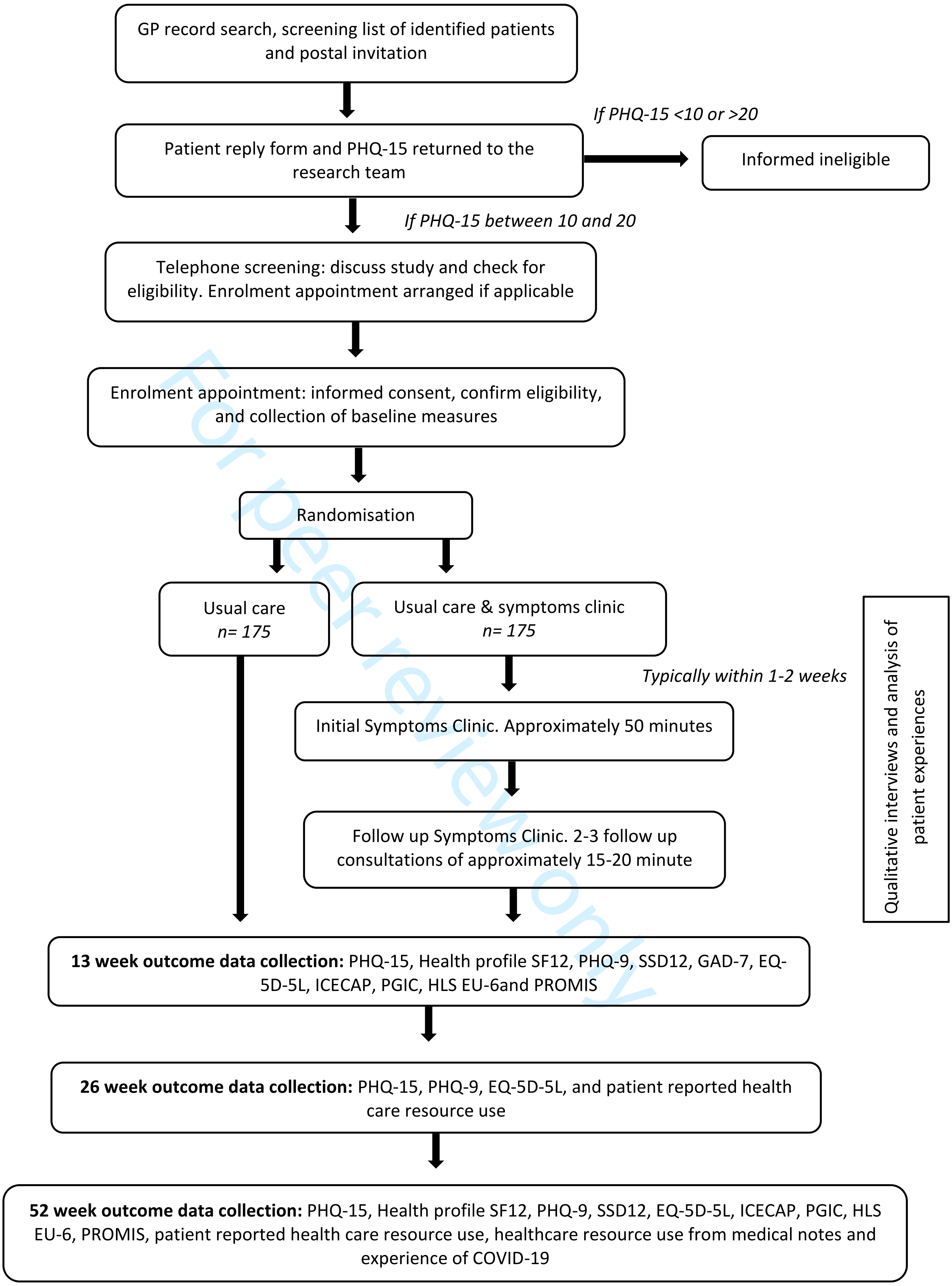

For peer review only

Supplementary material 1.

Search Strategy for MSS3

The search involve 4 steps; participants must meet inclusion criteria at each of the 4 stages

1. Inclusion based on age
2. Inclusion based on having no codes for serious medical conditions listed at any time
3. Inclusion if at least one code for a symptom disorder / syndrome (or repeat prescription for one in the last 10 years)
4. Inclusion if at least 2 referrals for specialist care in last 3 years.

Codes listed are Read CTV2

1. Age >18 & <70

2. AND NONE, EVER, OF

    a. Cancer (B. excluding B7, B8, BB)

    b. Diabetes mellitus (C10)

    c. Schizophrenic disorders (E10)

    d. Parkinson's disease(F12)

    e. Ischaemic heart disease (G3)

    f. Heart failure (G58)

    g. Cerebrovascular disease (G6)

    h. Rheumatoid arthritis and other inflammatory polyarthropathy (N04)

    i. Senile and presenile organic psychotic conditions (E00)

    j. Alcoholic psychoses (E02)

    k. Drug psychoses (E04)

    l. Other chronic organic psychoses (E04)

    m. [X]Organic, including symptomatic, mental disorders (Eu0)

    n. Other cerebral degenerations (F11)

    o. Housebound (13CA)

    p. [V]Palliative care (Zv57C)

    q. Palliative treatment (8BJ1)

    r. Terminal illness (1Z0)

For peer review only

s. X]Mental retardation (Eu7)

t. Mental retardation (E3)

u. [X]Specific developmental disorders of scholastic skills (Eu81)

3. AND EITHER ONE OR MORE IN THE LAST 10 YEARS OF

a. Psychalgia (E278)

b. [X]Tension type headache (F2626)

c. [D]Facial pain (R0400)

d. Temporomandibular joint disorders (J046)

e. History of irritable bowel syndrome (14CF)

f. Other female genital symptom (K58y)

g. [D]Pelvic and perineal pain R090G

h. Fibromyalgia N239

i. Fibromyalgia N248

j. [D]Non cardiac chest pain R065B

k. [D]Chronic intractable pain R00zC

l. [X]Dissociative [conversion] disorders Eu44

m. [X]Somatoform disorders Eu45

n. [X]Organic dissociative disorder Eu055

o. Hysteria E201 (excluding E2019, E201B, E201C)

p. [X]Mixed dissociative [conversion] disorders Eu447

q. [X]Unsp behav synd assoc with physiol disturb physical facts Eu5z

r. Functional gastrointestinal tract disorders NEC J52 excluding (J522,J523,J524)

s. Non epilepsy attack disorder EMISNQNO78

t. Medically unexplained symptoms 16T

u. Psychogenic vomiting NOS E2754

v. Functional vomiting J16y5

w. Persistent vomiting J162

For peer review only

x. 302641015

y. Other specified stomach function disorders J16y (excluding J16y0, J16y1, J16y2, J16y3,J16y4, J16yz)

z. [X]Nonorganic dyspareunia Eu256

aa. Physiological malfunction arising from mental factors E26

bb. Dysequilibrium syndrome SP3y8

OR ONE OR MORE IN THE LAST 3 YEARS OF REPEAT PRESRIPTION ISSUED FOR

cc. Hysocine butyl bromide

dd. Dicycloverine Hydrochloride

ee. Mebeverine Hydrochloride

ff. Dicyclomine

gg. Alverine citrate

4. AND

EITHER

Choose and book referral (8Hp) (>= 2 in last 3 years)

OR

Other referrals (see codes) (>= 2 in last 3 years) from any of the following

- Referral to physician 8H4 [except derm (8H43, 8H4S), geri (8H47, 8H4D, LD psych 8H4f, vasectomy 8h4i);
- Referral to surgeon 8H5 (except neuro 8H55; obstetric 8H57; Plastic 8H59)
- Priority cancer referral 8Hn (except skin 8Hn0; Breast 8Hn2; Haem 8Hn6

For peer review only

Supplementary material 2.

IRAS ID: 232038

Ethics reference: 18/NW/0422

Participant Identification Number:

CONSENT FORM

Title of Project: Multiple Symptoms Study 3

Researcher reads the following statement: Before I enter you in to the trial, I need to confirm with you that you understand what is involved and whether or not you agree to take part. I will be recording this process. Is that all right with you? (If yes, start recording and proceed as follows, refer to Baseline appointment guidance if required)

Please can you confirm your name and the date [pause for response]. I am going to read some statements to you. After each one, please answer 'yes' if you agree with the statement; or, 'no' if you do not agree with the statement.

Enter response (Y/N)

1. I confirm that I have read the information sheet dated.................... (version...........) for the above study. I have had the opportunity to consider the information, ask questions and have had these answered satisfactorily. Y/N

2. I understand that it is my choice to participate and that I am free to leave the study at any time without giving any reason, and without my medical care or legal rights being affected. Y/N

3. I understand that relevant sections of my medical notes and data collected during the study may be looked at by research staff involved in the study, from regulatory authorities or from the NHS Trust, where it is relevant to my taking part in this research. I give permission for these individuals to have access to my records. Y/N

4. I agree that the research team can use the information that I have provided in the completed questionnaire/s for the purposes of this study. Y/N

5. I agree that if I am allocated to receive the Symptoms Clinic that these consultations can be audio recorded. Y/N

6. I understand that the information collected about me may be used to support other research in the Y/N

When completed: 1 for participant; 1 for researcher site file

MSS3 Consent form v3.0, 09.07.2020

For peer review only

Supplementary material 2.

future, and may be shared anonymously with other researchers.

7. I agree to my General Practitioner being informed of my participation in the study and relevant information about me being exchanged between my GP and the research team. Y/N

8. I agree that a copy of this consent form and other study documents can be posted to the University of Sheffield for monitoring purposes Y/N

9. I agree to take part in the above study. Y/N

Optional Statements

10. I am happy for a member of the research team to contact me about taking part in an interview about my experience in this study. (Please note, this is entirely optional and does not affect your involvement in the rest of the study). Y/N

11. I agree that members of the research team can approach me at a later date to ask me if I wish to participate in future studies similar to this project. (Please note, this is entirely optional and does not affect your involvement in the rest of the study). Y/N

Declaration

I confirm that I have read each statement to the participant and they have given full consent to take part.

Name of person taking audio consent	Date	Signature

When completed: 1 for participant; 1 for researcher site file

MSS3 Consent form v3.0, 09.07.2020

For peer review only

Supplementary material 3.

The Symptoms Clinic Intervention

The Recognition, Explanation, Action and Learning (REAL) intervention was structured as follows.

Recognition takes place during the history-taking phase of the intervention. It includes explicit recognition of, and belief in, the reality and legitimacy of the patient's experience. It also includes explicit recognition that persistent physical symptoms are within the scope of these medical consultations and can be understood without recourse to primary psychological causes. Recognition also seeks to build therapeutic alliance between ER-GP and patient.

Explanation seeks to propose and negotiate explanations for symptoms in terms of body physiology, and sensory signal processing. Explanations seek to portray symptoms as understandable (in contrast to the idea of "medically unexplained symptoms") adaptive responses in body processes. ER-GPs delivering the Symptoms Clinic are encouraged to use the names of syndromes such as irritable bowel syndrome and fibromyalgia where criteria for these are met. However, explanations aim to provide mechanisms for the symptoms which extend beyond simply attributing a symptom to a syndrome.

Action to manage symptoms is proposed after explanations have been offered and negotiated. Actions can include attending to the body, thoughts and emotions, and the personal or social environment. Body-focused actions include breathing techniques (diaphragmatic breathing, slow paced breathing), relaxation, sensory grounding and simple guided imagery[20]. Actions around thoughts and emotions range include addressing catastrophic or symptom-focused thinking. Actions around behaviors include pacing, effective rest and behavioural activation (where dysphoria was an issue). For some patients recommended action includes taking steps to engage with psychological therapies – for instance where trauma emerges as part of the explanation.

Learning comes from the participant implementing agreed actions and evaluating the impact of them on their symptoms. Learning also relates to the importance of summing up sessions and the course of treatment with key take-homes. This is also facilitated by letters to the patient's usual GP (copied to the patient) after the first and final consultation summarizing some of the key points covered in the clinic.

The Symptoms Clinic intervention is described in a manual provided to the ER-GPs; however it is designed to be delivered flexibly and in a person-centred way which allows the clinician considerable freedom to focus on aspects of the patient's problem that they deem most appropriate and in forms of words they personally feel comfortable with.

For peer review only

SPIRIT

STANDARD PROTOCOL ITEMS: RECOMMENDATIONS FOR INTERVENTIONAL TRIALS

SPIRIT 2013 Checklist: Recommended items to address in a clinical trial protocol and related documents*

Section/item	Item No	Description	Page
**Administrative information**			
Title	1	Descriptive title identifying the study design, population, interventions, and, if applicable, trial acronym	**1**
Trial registration	2a	Trial identifier and registry name. If not yet registered, name of intended registry	**2**
	2b	All items from the World Health Organization Trial Registration Data Set	**1**
Protocol version	3	Date and version identifier	**1**
Funding	4	Sources and types of financial, material, and other support	**14**
Roles and responsibilities	5a	Names, affiliations, and roles of protocol contributors	**1 & 13**
	5b	Name and contact information for the trial sponsor	**12**
	5c	Role of study sponsor and funders, if any, in study design; collection, management, analysis, and interpretation of data; writing of the report; and the decision to submit the report for publication, including whether they will have ultimate authority over any of these activities	**12 & 14**
	5d	Composition, roles, and responsibilities of the coordinating centre, steering committee, endpoint adjudication committee, data management team, and other individuals or groups overseeing the trial, if applicable (see Item 21a for data monitoring committee)	**12**
**Introduction**			
Background and rationale	6a	Description of research question and justification for undertaking the trial, including summary of relevant studies (published and unpublished) examining benefits and harms for each intervention	**3 & 4**
	6b	Explanation for choice of comparators	**4**
Objectives	7	Specific objectives or hypotheses	**4**
Trial design	8	Description of trial design including type of trial (eg, parallel group, crossover, factorial, single group), allocation ratio, and framework (eg, superiority, equivalence, noninferiority, exploratory)	**4**

For peer review only

Methods: Participants, interventions, and outcomes			Page
Study setting	9	Description of study settings (eg, community clinic, academic hospital) and list of countries where data will be collected. Reference to where list of study sites can be obtained	**4 & 5**
Eligibility criteria	10	Inclusion and exclusion criteria for participants. If applicable, eligibility criteria for study centres and individuals who will perform the interventions (eg, surgeons, psychotherapists)	**5**
Interventions	11a	Interventions for each group with sufficient detail to allow replication, including how and when they will be administered	**7 & Suppl. 3**
	11b	Criteria for discontinuing or modifying allocated interventions for a given trial participant (eg, drug dose change in response to harms, participant request, or improving/worsening disease)	**9**
	11c	Strategies to improve adherence to intervention protocols, and any procedures for monitoring adherence (eg, drug tablet return, laboratory tests)	**7 & 9**
	11d	Relevant concomitant care and interventions that are permitted or prohibited during the trial	**6**
Outcomes	12	Primary, secondary, and other outcomes, including the specific measurement variable (eg, systolic blood pressure), analysis metric (eg, change from baseline, final value, time to event), method of aggregation (eg, median, proportion), and time point for each outcome. Explanation of the clinical relevance of chosen efficacy and harm outcomes is strongly recommended	**8**
Participant timeline	13	Time schedule of enrolment, interventions (including any run-ins and washouts), assessments, and visits for participants. A schematic diagram is highly recommended (see Figure)	**6**
Sample size	14	Estimated number of participants needed to achieve study objectives and how it was determined, including clinical and statistical assumptions supporting any sample size calculations	**9**
Recruitment	15	Strategies for achieving adequate participant enrolment to reach target sample size	**5 & 6**
**Methods: Assignment of interventions (for controlled trials)**			
Allocation:			
Sequence generation	16a	Method of generating the allocation sequence (eg, computer-generated random numbers), and list of any factors for stratification. To reduce predictability of a random sequence, details of any planned restriction (eg, blocking) should be provided in a separate document that is unavailable to those who enrol participants or assign interventions	**6**

For peer review only - http://bmjopen.bmj.com/site/about/guidelines.xhtml

Allocation concealment mechanism	16b	Mechanism of implementing the allocation sequence (eg, central telephone; sequentially numbered, opaque, sealed envelopes), describing any steps to conceal the sequence until interventions are assigned	**6**
Implementation	16c	Who will generate the allocation sequence, who will enrol participants, and who will assign participants to interventions	**6**
Blinding (masking)	17a	Who will be blinded after assignment to interventions (eg, trial participants, care providers, outcome assessors, data analysts), and how	**6**
	17b	If blinded, circumstances under which unblinding is permissible, and procedure for revealing a participant's allocated intervention during the trial	**8**
**Methods: Data collection, management, and analysis**			
Data collection methods	18a	Plans for assessment and collection of outcome, baseline, and other trial data, including any related processes to promote data quality (eg, duplicate measurements, training of assessors) and a description of study instruments (eg, questionnaires, laboratory tests) along with their reliability and validity, if known. Reference to where data collection forms can be found, if not in the protocol	**6 & 8**
	18b	Plans to promote participant retention and complete follow-up, including list of any outcome data to be collected for participants who discontinue or deviate from intervention protocols	**8 & 9**
Data management	19	Plans for data entry, coding, security, and storage, including any related processes to promote data quality (eg, double data entry; range checks for data values). Reference to where details of data management procedures can be found, if not in the protocol	**8**
Statistical methods	20a	Statistical methods for analysing primary and secondary outcomes. Reference to where other details of the statistical analysis plan can be found, if not in the protocol	**9 - 12**
	20b	Methods for any additional analyses (eg, subgroup and adjusted analyses)	**9 & 10**
	20c	Definition of analysis population relating to protocol non-adherence (eg, as randomised analysis), and any statistical methods to handle missing data (eg, multiple imputation)	**9 & 10**
**Methods: Monitoring**			
Data monitoring	21a	Composition of data monitoring committee (DMC); summary of its role and reporting structure; statement of whether it is independent from the sponsor and competing interests; and reference to where further details about its charter can be found, if not in the protocol. Alternatively, an explanation of why a DMC is not needed	**12**

	21b	Description of any interim analyses and stopping guidelines, including who will have access to these interim results and make the final decision to terminate the trial	**N/A**
Harms	22	Plans for collecting, assessing, reporting, and managing solicited and spontaneously reported adverse events and other unintended effects of trial interventions or trial conduct	**12**
Auditing	23	Frequency and procedures for auditing trial conduct, if any, and whether the process will be independent from investigators and the sponsor	**12**
**Ethics and dissemination**			
Research ethics approval	24	Plans for seeking research ethics committee/institutional review board (REC/IRB) approval	**12**
Protocol amendments	25	Plans for communicating important protocol modifications (eg, changes to eligibility criteria, outcomes, analyses) to relevant parties (eg, investigators, REC/IRBs, trial participants, trial registries, journals, regulators)	**13**
Consent or assent	26a	Who will obtain informed consent or assent from potential trial participants or authorised surrogates, and how (see Item 32)	**6**
	26b	Additional consent provisions for collection and use of participant data and biological specimens in ancillary studies, if applicable	**N/A**
Confidentiality	27	How personal information about potential and enrolled participants will be collected, shared, and maintained in order to protect confidentiality before, during, and after the trial	**8**
Declaration of interests	28	Financial and other competing interests for principal investigators for the overall trial and each study site	**13 & 14**
Access to data	29	Statement of who will have access to the final trial dataset, and disclosure of contractual agreements that limit such access for investigators	**13**
Ancillary and post-trial care	30	Provisions, if any, for ancillary and post-trial care, and for compensation to those who suffer harm from trial participation	**NA**
Dissemination policy	31a	Plans for investigators and sponsor to communicate trial results to participants, healthcare professionals, the public, and other relevant groups (eg, via publication, reporting in results databases, or other data sharing arrangements), including any publication restrictions	**13**
	31b	Authorship eligibility guidelines and any intended use of professional writers	**13**
	31c	Plans, if any, for granting public access to the full protocol, participant-level dataset, and statistical code	**13**

For peer review only - http://bmjopen.bmj.com/site/about/guidelines.xhtml

Appendices

Informed consent materials	32	Model consent form and other related documentation given to participants and authorised surrogates	**6 & Suppl. 2**
Biological specimens	33	Plans for collection, laboratory evaluation, and storage of biological specimens for genetic or molecular analysis in the current trial and for future use in ancillary studies, if applicable	**N/A**

*It is strongly recommended that this checklist be read in conjunction with the SPIRIT 2013 Explanation & Elaboration for important clarification on the items. Amendments to the protocol should be tracked and dated. The SPIRIT checklist is copyrighted by the SPIRIT Group under the Creative Commons "Attribution-NonCommercial-NoDerivs 3.0 Unported" license.

For peer review only
